# Temporal Trends in End-Tidal Capnography and Outcomes in Out-of-Hospital Cardiac Arrest: A Secondary Analysis of a Randomized Clinical Trial

**DOI:** 10.1001/jamanetworkopen.2024.19274

**Published:** 2024-07-05

**Authors:** Michelle M. J. Nassal, Andoni Elola, Elisabete Aramendi, Xabier Jaureguibeitia, Jonathan R. Powell, Ahamed Idris, Banu Priya Raya Krishnamoorthy, Mohamud R. Daya, Tom P. Aufderheide, Jestin N. Carlson, Shannon W. Stephens, Ashish R. Panchal, Henry E. Wang

**Affiliations:** 1 Department of Emergency Medicine, The Ohio State University, Columbus; 2 Department of Electronic Technology, BioRes Group, University of the Basque Country, UPV/EHU, Bilbao, Spain; 3 Department of Communication Engineering, BioRes Group, University of the Basque Country, UPV/EHU, Bilbao, Spain; 4 Department of Emergency Medicine, University of Texas Southwestern Medical Center, Dallas; 5 Department of Computer Science and Engineering, Emergency Medicine, The Ohio State University, Columbus; 6 Department of Emergency Medicine, Oregon Health & Science University, Portland; 7 Department of Emergency Medicine, Medical College of Wisconsin, Milwaukee; 8 Department of Emergency Medicine, University of Pittsburgh, Pittsburgh, Pennsylvania; 9 Department of Surgery, The University of Alabama, Birmingham

## Abstract

**Importance:**

While widely measured, the time-varying association between exhaled end-tidal carbon dioxide (EtCO_2_) and out-of-hospital cardiac arrest (OHCA) outcomes is unclear.

**Objective:**

To evaluate temporal associations between EtCO_2_ and return of spontaneous circulation (ROSC) in the Pragmatic Airway Resuscitation Trial (PART).

**Design, Setting, and Participants:**

This study was a secondary analysis of a cluster randomized trial performed at multicenter emergency medical services agencies from the Resuscitation Outcomes Consortium. PART enrolled 3004 adults (aged ≥18 years) with nontraumatic OHCA from December 1, 2015, to November 4, 2017. EtCO_2_ was available in 1172 cases for this analysis performed in June 2023.

**Interventions:**

PART evaluated the effect of laryngeal tube vs endotracheal intubation on 72-hour survival. Emergency medical services agencies collected continuous EtCO_2_ recordings using standard monitors, and this secondary analysis identified maximal EtCO_2_ values per ventilation and determined mean EtCO_2_ in 1-minute epochs using previously validated automated signal processing. All advanced airway cases with greater than 50% interpretable EtCO_2_ signal were included, and the slope of EtCO_2_ change over resuscitation was calculated.

**Main Outcomes and Measures:**

The primary outcome was ROSC determined by prehospital or emergency department palpable pulses. EtCO_2_ values were compared at discrete time points using Mann-Whitney test, and temporal trends in EtCO_2_ were compared using Cochran-Armitage test of trend. Multivariable logistic regression was performed, adjusting for Utstein criteria and EtCO_2_ slope.

**Results:**

Among 1113 patients included in the study, 694 (62.4%) were male; 285 (25.6%) were Black or African American, 592 (53.2%) were White, and 236 (21.2%) were another race; and the median (IQR) age was 64 (52-75) years. Cardiac arrest was most commonly unwitnessed (n = 579 [52.0%]), nonshockable (n = 941 [84.6%]), and nonpublic (n = 999 [89.8%]). There were 198 patients (17.8%) with ROSC and 915 (82.2%) without ROSC. Median EtCO_2_ values between ROSC and non-ROSC cases were significantly different at 10 minutes (39.8 [IQR, 27.1-56.4] mm Hg vs 26.1 [IQR, 14.9-39.0] mm Hg; *P* < .001) and 5 minutes (43.0 [IQR, 28.1-55.8] mm Hg vs 25.0 [IQR, 13.3-37.4] mm Hg; *P* < .001) prior to end of resuscitation. In ROSC cases, median EtCO_2_ increased from 30.5 (IQR, 22.4-54.2) mm HG to 43.0 (IQR, 28.1-55.8) mm Hg (*P *for trend < .001). In non-ROSC cases, EtCO_2_ declined from 30.8 (IQR, 18.2-43.8) mm Hg to 22.5 (IQR, 12.8-35.4) mm Hg (*P *for trend < .001). Using adjusted multivariable logistic regression with slope of EtCO_2_, the temporal change in EtCO_2_ was associated with ROSC (odds ratio, 1.45 [95% CI, 1.31-1.61]).

**Conclusions and Relevance:**

In this secondary analysis of the PART trial, temporal increases in EtCO_2_ were associated with increased odds of ROSC. These results suggest value in leveraging continuous waveform capnography during OHCA resuscitation.

**Trial Registration:**

ClinicalTrials.gov Identifier: NCT02419573

## Key Points

**Question:**

Is there an association between end-tidal capnography changes over resuscitation and outcomes from out-of-hospital cardiac arrest?

**Findings:**

In this secondary analysis of 1113 patients in the Pragmatic Airway Resuscitation Trial, temporal increases in end-tidal capnography over resuscitation were associated with return-of-spontaneous circulation in out-of-hospital cardiac arrest.

**Meaning:**

These results suggest the importance of dynamic time-varying end-tidal capnography, which may be leveraged by clinicians in guiding resuscitation decisions.

## Teaser

This secondary analysis evaluates temporal associations between end-tidal capnography and return of spontaneous circulation among patients with out-of-hospital cardiac arrest in the Pragmatic Airway Resuscitation Trial.

## Introduction

Cardiac arrest is a leading cause of death globally.^1,2,3^ Despite decades of research in out-of-hospital cardiac arrest (OHCA), survival has remained low.^1^ To improve survival, both the International Liaison Committee on Resuscitation (ILCOR) and American Heart Association prehospital recommendations have emphasized the importance of 9-1-1 activation, rapid bystander cardiopulmonary resuscitation, early defibrillation, and provision of high-quality cardiopulmonary resuscitation (CPR).^4,5^ In addition, recent studies have highlighted the importance of high-quality ventilation for improved survival and the need for ventilation monitoring during OHCA.^6^

Guideline recommendations encourage use of end-tidal carbon dioxide (EtCO_2_) capnography during resuscitation from OHCA for confirmation of advanced airway placement as well as monitoring progress of resuscitation.^4,6^ However, the practical application of capnography for the latter goal is unclear.^7,8^ Prior studies evaluated the associations between single discrete time-point EtCO_2_ values with return of spontaneous circulation (ROSC) detection or termination of resuscitation rules but sensitivities were as low as 20% to 33%.^9,10^ The most comprehensive data analysis was compiled by an ILCOR systematic review which stated that continuous EtCO_2_ capnography through trending EtCO_2_ may be a better predictor of cardiac arrest outcomes.^8,11,12,13,14,15^ To date, limited data has been presented to support this approach.

The dynamic variations in EtCO_2_ capnometry during resuscitation and the association with outcomes remains unknown. Our objective was to determine the association between temporal trends of EtCO_2_ and ROSC in the Pragmatic Airway Resuscitation Trial (PART).

## Methods

### Study Design, Setting, and Participants

This was a secondary analysis of EtCO_2_ capnography waveforms from the PART trial.^16^ The PART trial enrolled adults (age ≥18 years) with nontraumatic out-of-hospital cardiac arrest from 27 emergency medical services (EMS) agencies from 5 communities of the Resuscitation Outcomes Consortium. PART, a cluster randomized trial, assigned adult OHCA to strategies of laryngeal tube insertion or endotracheal intubation airway management. Exclusion criteria consisted of patients less than 18 years of age, pregnant people, prisoners, and traumatic OHCAs. The trial enrolled patients from December 1, 2015, through November 4, 2017. Race and ethnicity in PART was reported from EMS agencies (and included the following categories: Black or African American, White, and other [American Indian or Alaska Native, Asian, Hispanic, Pacific Islander, other, and unknown]). The trial protocol is available in Supplement 1. For this post hoc analysis, we included only participants in whom an advanced airway was successfully placed and with continuous capnography data available. The institutional review board of The Ohio State University approved this retrospective secondary analysis of the parent trial and informed consent was waived because patients had an emergent condition and were unable to consent in time for treatment (in accordance with 21 CFR 50.24). This study followed the Consolidated Standards of Reporting Trials (CONSORT) reporting guideline.

### Measures

The primary measure was EtCO_2_ over the course of resuscitation from initial placement through either ROSC or termination of resuscitation. EMS agencies collected continuous EtCO_2_ capnography waveforms using portable cardiac defibrillator monitors as part of their advanced airway standard of care. The cardiac monitors used in this study were the LifePak 15 series (Physio-Control), the X-series (ZOLL Medical Corporation) and the MRx series (Philips Healthcare).

We identified maximal EtCO_2_ values for each ventilation using previously validated automated signal processing.^17^ Import and analysis of capnography waveforms were accomplished using MATLAB (Mathworks) and a custom graphical user interface (GUI).^18,19^ The algorithm detects maximal EtCO_2_ values per ventilation. We determined mean EtCO_2_ in 1-minute epochs. We included all cases with greater than 50% interpretable EtCO_2_ signal in at least 1 of the epochs. We also provide histogram plots of the change in capnography from initial to end of resuscitation and box plots displaying distribution of slope calculations to evaluate individualized change in capnography.

### Outcomes

Our primary outcome of interest was ROSC, which was determined by clinical evaluation of palpable pulses as marked in the parent trial by EMS clinicians or physicians at the receiving emergency department. Secondary outcomes included survival to 72 hours after cardiac arrest. We also separated nonsurvivors (including those who obtained ROSC) and survivors based on clinical information marked in the parent trial by hospital physicians.

### Statistical Analysis

We planned to evaluate temporal changes in EtCO_2_ in relation to ROSC. We divided cases into ROSC or non-ROSC and survivors or nonsurvivors for analysis. We included the time from ROSC or cessation of resuscitation efforts defined as last chest compression and up to the previous 20 minutes of resuscitation for figure presentations. Therefore, time in this analysis is marked by negative numbers where −20 minutes would represent initial or early EtCO_2_ values on EMS arrival (eAppendix 1 in Supplement 2).

We compared discrete time points between ROSC vs non-ROSC and survivors vs nonsurvivors using the Mann-Whitney test. We determined the association between temporal trends in EtCO_2_ using Cochran-Armitage test of trend. The slope of EtCO_2_ was calculated by change in EtCO_2_ over sequential minutes available during the resuscitation (mm Hg/min). Tests were 2-sided and *P* < .05 was considered significant. Finally, we performed an adjusted multivariable logistic regression model for outcomes adjusted for the slope of EtCO_2_, age, sex, witnessed cardiac arrest (bystander, EMS witnessed, unwitnessed), bystander CPR (yes/no), initial ECG rhythm (shockable vs nonshockable), public location, chest compression rate (within American Heart Association [AHA] recommendations of 100-120 [yes/no]), chest compression depth (within AHA recommendations of 5-6 cm [yes/no]), successful airway placed and epinephrine given (yes/no). Chest compression fraction within AHA recommended rates (>0.6) was achieved in 99.6% of all cases so this covariate was omitted.

In a sensitivity analysis to account for potential ventilation quality effects on capnography, we repeated analysis using ventilations only within AHA recommendations of 6 to 12 breaths per minute. As length of resuscitation time may affect modeling, we performed a stratified multivariate adjusted regression model considering less than 10 minutes resuscitation to be short resuscitation and greater than 10 minutes resuscitation to be prolonged resuscitation. We also repeated the analysis using generalized estimated equations to account for the randomized cluster trial design. We considered multicollinearity within our models using a variance inflation factor greater than 10. We assessed goodness-of-fit testing using Hosmer-Lemeshow statistics. We considered our models to have acceptable discrimination if the area under the receiver operating characteristic curve (AUC) was at least 0.70; excellent discrimination if AUC was at least 0.8, and outstanding discrimination if AUC was at least 0.9.^20^ Analysis was conducted using Stata version 16.0 (Stata Inc) from X to Y.

## Results

**Figure 1.**
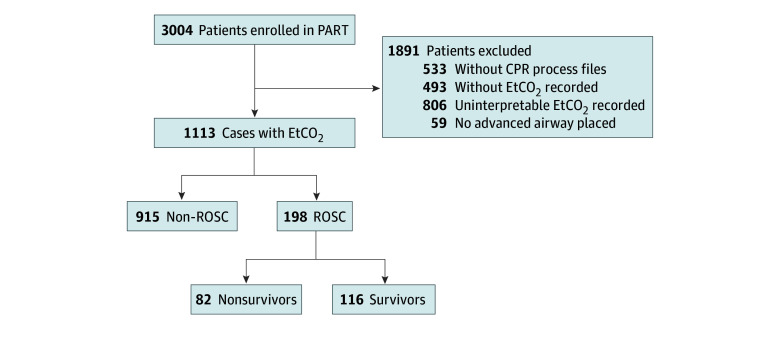
Cases From the Pragmatic Airway Resuscitation Trial (PART) Included in This Study After exclusion of cases without CPR process files, less than 50% interpretable EtCO_2_ capnography in at least 1 minute epoch and no advanced airway placed; there were 1113 cases available for analysis. CPR indicates cardiopulmonary resuscitation; EtCO_2_, end-tidal carbon dioxide; ROSC, return of spontaneous circulation.

Of the 3004 patients included in PART, 1113 had an advanced airway with EtCO_2_ capnography waveforms that met initial quality assessments. ROSC occurred in 196 (17.8%) and 116 (10.4%) survived 72 hours post cardiac arrest (Figure 1). Among the 1113 patients in our cohort, 694 (62.4%) were male and 419 (37.6%) were female; 285 (25.6%) were Black or African American, 592 (53.2%) were White, and 236 (21.2%) were other race or ethnicity; and the median (IQR) age was 64 (52-75) years (Table 1). The most common cardiac arrest was unwitnessed (n = 579 [52.0%]), nonshockable (n = 941 [84.6%]), and nonpublic (n = 999 [89.8%]).

**Table 1.** Demographics of the Included Population

Individual characteristics	Participants, No. (%) (N = 1113)
Age, median (IQR), y	64 (52-75)
Sex	
Male	694 (62.4)
Female	419 (37.6)
Race and ethnicity	
Black or African American	285 (25.6)
White	592 (53.2)
Other^a^	236 (21.2)
Location of arrest	
Public	113 (10.2)
Nonpublic	999 (89.8)
Missing	1 (0.0)
Witnessed status	
Unwitnessed	579 (52.0)
Bystander witnessed	327 (29.4)
911 Responder witnessed	105 (9.4)
Missing	102 (9.2)
Initial rhythm	
Shockable	172 (15.5)
Nonshockable	941 (84.6)
Bystander CPR	556 (50.0)
Bystander AED	121 (11.0)
ROSC	198 (17.8)
72 h Survival	116 (10.4)

Abbreviations: AED, automatic external defibrillator; CPR, cardiopulmonary resuscitation; ROSC, return of spontaneous circulation.

^a^ Other race and ethnicity included American Indian or Alaska Native, Asian, Hispanic, Pacific Islander, other, and unknown.

**Figure 2.**
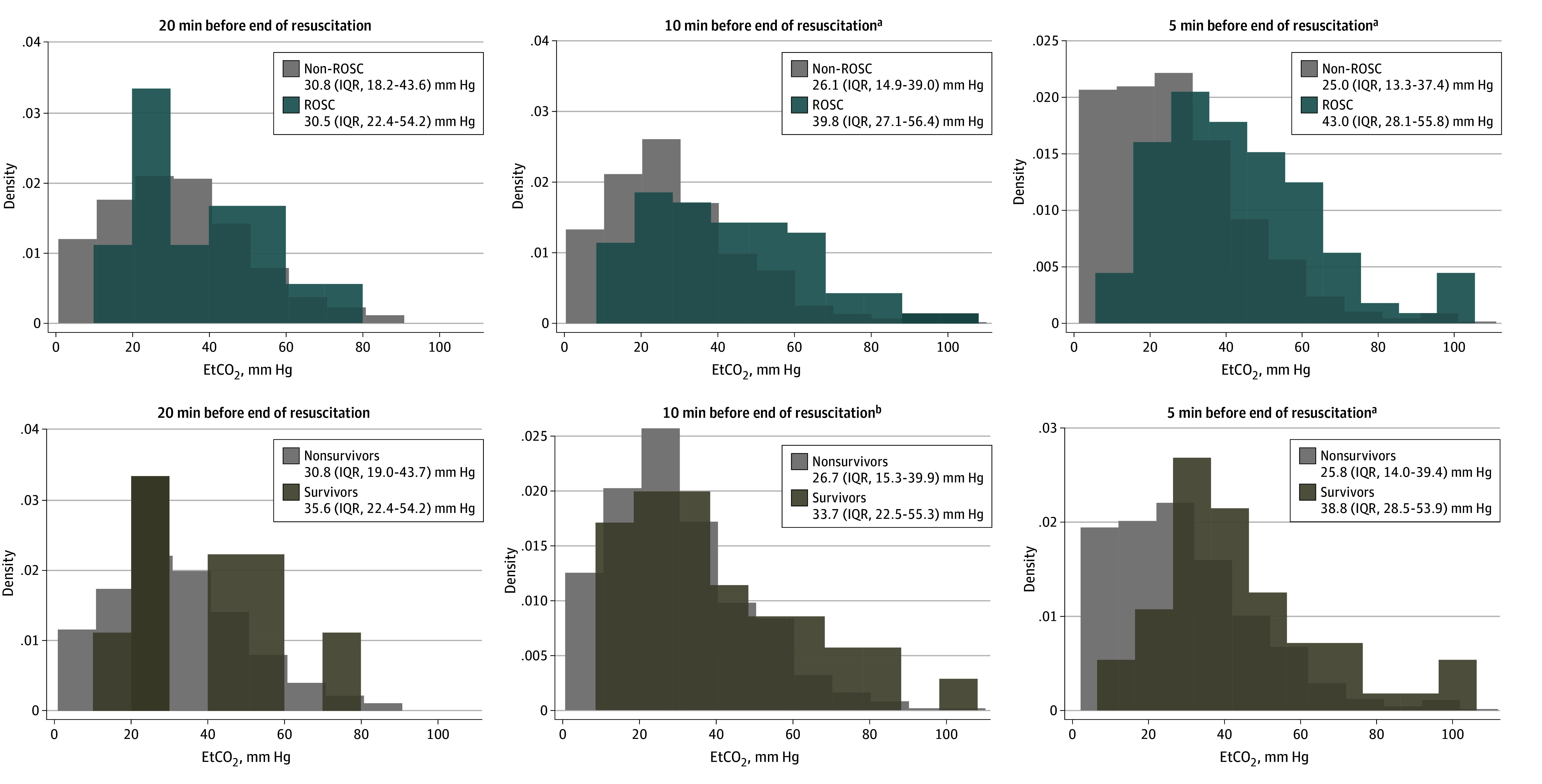
Histograms of End-Tidal Carbon Dioxide (EtCO_2_) Levels at Discrete Time Points in the Resuscitation (20, 10, 5 minutes) Prior to End of the Event Return of spontaneous circulation (ROSC) vs non-ROSC patients (top row) and survivors vs nonsurvivors (bottom row). Median (IQR) EtCO_2_ levels are displayed in color-coordinated boxes in top right of each graph. ^a^*P* < .05. ^b^*P* < .01.

Mean (SD) duration of resuscitation was 24 (10) minutes in ROSC cases and 19 (9) in non-ROSC cases. EtCO_2_ values for each individual case over 10 minutes of resuscitation are shown (eAppendix 2 in Supplement 2). The change in EtCO_2_ values over resuscitation and distribution of slopes per group are also shown (eAppendix 2 in Supplement 2). Median EtCO_2_ values in patients without ROSC were different from patients with ROSC at 10 minutes (without ROSC: 26.1 [IQR, 14.9-39.0] mm Hg vs with ROSC: 39.8 [IQR, 27.1-56.4] mm Hg; *P* < .001) and 5 minutes (without ROSC: 25.0 [IQR, 13.3-37.4] mm Hg vs with ROSC: 43.0 [IQR, 28.1-55.8] mm Hg; *P* < .001) prior to end of resuscitation (Figure 2). Similarly, median EtCO_2_ values in nonsurvivors were significantly different than survivors at 10 minutes (nonsurvivors: 26.7 [IQR, 15.3-39.9] mm Hg vs survivors: 33.7 [IQR, 22.5-55.3] mm Hg; *P* = .01) and 5 minutes (25.8 [IQR, 14.0-39.3] mm Hg vs 38.8 [IQR, 28.5-53.9] mm Hg, *P* < .001) prior to the end of resuscitation. Median EtCO_2_ values at 20 minutes prior to the end of resuscitation did not differentiate groups in either ROSC vs non-ROSC (without ROSC: 30.8 [IQR, 18.2-43.8] mm Hg vs with ROSC: 30.5 [IQR, 22.4-54.2] mm Hg; *P* = .40) or survivors vs nonsurvivors (35.6 [IQR, 22.4-54.2] mm Hg vs 30.8 [IQR, 19.0-43.7] mm Hg; *P* = .37; Figure 2).

**Figure 3.**
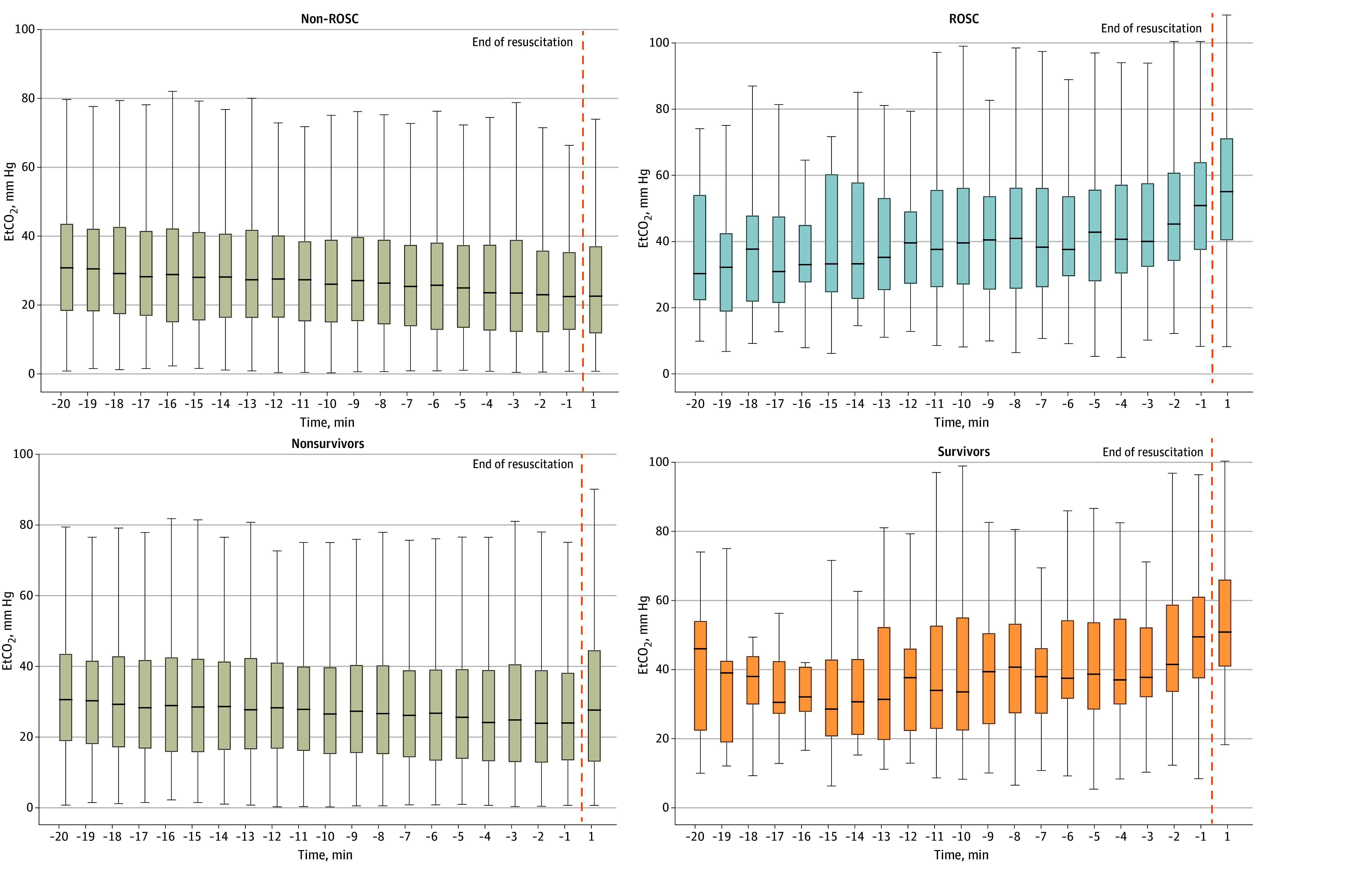
Summary End-Tidal Carbon Dioxide (EtCO_2_) Over Resuscitation Until End of the Event Return of spontaneous circulation (ROSC) vs non-ROSC cases are shown in the top row. Survivors vs nonurvivors are shown in the bottom row. The center line in each box indicates the 50th percentile (median) of that category’s EtCO_2_ value. The bottom of each box indicates the 25th percentile, and the top of each box indicates the 75th percentile. The whisker below the box is the lower adjacent value (equal to the 25th percentile minus 1.5 times the IQR). The whisker above the box is the upper adjacent value (equal to the 75th percentile plus 1.5 times the IQR).

The trend of EtCO_2_ values were different among groups over resuscitation. In ROSC cases, median EtCO_2_ increased from 30.5 (IQR, 22.4-54.2) mm Hg to 51.0 (IQR, 37.6-64.1) mm Hg over resuscitation (*P* for trend = .001). In non-ROSC cases, median EtCO_2_ decreased from 30.8 (IQR, 18.2-43.8) mm Hg to 22.5 (IQR, 12.8-35.4) mm Hg (*P* for trend = .001) (Figure 3). Similarly, in survivors, EtCO_2_ increased from 33.7 (IQR, 22.5-55.3) mm Hg to 49.5 (IQR, 37.6-61.3) mm Hg from 10 minutes to 1 minute prior to end of resuscitation (*P* for trend = .001) (Figure 3).

Adjusting for Utstein variables including age, sex, public location, bystander witnessed status, bystander CPR, initial rhythm, chest compression rate, chest compression depth, and epinephrine given; the slope of EtCO_2_ change over resuscitation was associated with both ROSC and survival (logistic regression *P* < .001) (Table 2). Discrimination for ROSC (0.78 [95% CI, 0.73-0.80]) was acceptable and for survival (0.82 [95% CI, 0.77-0.85]) was excellent. Goodness-of-fit testing statistics were acceptable for ROSC (0.46) and survival (0.49). As a sensitivity analysis using only AHA-recommended ventilation rates for inclusion in a multivariate-adjusted regression model, the slope of EtCO_2_ change over resuscitation remained associated with ROSC (odds ratio [OR], 1.22 [95% CI, 1.11-1.34]) and survival (OR, 1.19 [95% CI, 1.06-1.34]). As length of resuscitation also may differentially contribute to outcomes we performed another stratified analysis by resuscitation time. Slope of EtCO_2_ change over resuscitation remains associated with outcomes in both short ROSC (OR, 1.27 [95% CI, 1.12-1.43]) and prolonged ROSC (OR, 1.53 [95% CI, 1.29-1.83]) resuscitations (eAppendix 3 in Supplement 2). Accounting for trial design did not affect modeling associations (eAppendix 4 in Supplement 2).

**Table 2.** Multivariate Adjusted Logistic Regression Model for Outcomes

Variable	OR (95% CI)	
	ROSC	Survival
Slope of EtCO_2_	1.45 (1.31-1.61)	1.33 (1.20-1.45)
Age	0.99 (0.98-1.00)	0.98 (0.97-0.99)
Sex		
Male	0.89 (0.61-1.30)	0.88 (0.55-1.42)
Female	1 [Reference]	1 [Reference]
Public location	1.85 (1.09-3.15)	1.86 (1.02-3.40)
Shockable rhythm	1.78 (1.15-2.78)	2.69 (1.62-4.46)
Bystander CPR	0.85 (0.57-1.26)	0.82 (0.49-1.36)
Bystander witnessed	2.94 (1.95-4.43)	4.24 (2.43-7.38)
EMS witnessed	3.93 (2.24-6.91)	5.72 (2.80-11.70)^a^
Epinephrine	0.90 (0.25-3.29)	1.26 (0.25-6.38)
Chest compression rate within 100-120	1.07 (0.68-1.71)	0.60 (0.36-1.02)
Chest compression depth within 5 to 6 cm	1.02 (0.71-1.46)	0.88 (0.55-1.39)
Airway successfully placed tube		
Endotracheal tube	1.08 (0.72-1.62)	0.85 (0.51-1.43)
Laryngeal tube	1 [Reference]	1 [Reference]

Abbreviations: CPR, cardiopulmonary resuscitation; EMS, emergency medical services; EtCO_2_, end-tidal carbon dioxide; OR, odds ratio; ROSC, return of spontaneous circulation.

## Discussion

Dynamic temporal changes in continuous EtCO_2_ waveform capnography have tremendous potential in resuscitation. In this secondary analysis we found that while early discrete EtCO_2_ values did not differentiate between outcomes; temporal increases in EtCO_2_ over time were associated with both ROSC and survival. These findings emphasize the importance of dynamic time-varying EtCO_2_ capnography which may be leveraged in guiding resuscitation decisions, especially for termination or transportation decisions in the prehospital space.

Prior studies used simpler approaches, including discrete time point EtCO_2_ values, to characterize capnography in OHCA resuscitation.^8,11,21,22^ Initial EtCO_2_ values below 10 mm Hg have correlated with poor outcomes and values above 20 mm Hg correlated with improved outcomes.^23^ Early EtCO_2_ values were unexpectedly higher in our study. Among patients without ROSC, median EtCO_2_ values were 30.8 mm Hg. Furthermore, within the ROSC groups, 3 cases had EtCO_2_ values below 10 mm Hg. Previous studies have also shown 100% sensitivity with EtCO_2_ values below 14 mm Hg after 20 minutes of resuscitation. Many (40%) of our patients without ROSC had higher values than 14 mm Hg toward the end of resuscitation.^21^ These emphasize the variability and challenges associated with interpreting discrete EtCO_2_ values during resuscitation.^24^

Continuous capnography offers advantages in that it can account for waveform variability and allows for monitoring change during resuscitation.^8^ Through automated signal processing,^17,19,25^ vital EtCO_2_ information such as EtCO_2_ value change and rate of change can be quickly obtained and correlated with outcomes. Similar to our findings, 2 studies found that the absence of decreasing EtCO_2_ from initial to final EtCO_2_ value was associated with achieving ROSC in OHCA.^11,15^ Our study shows the benefit in leveraging capnography over the resuscitation rather than discrete time points. Collectively, these works encourage the use of dynamic changes in EtCO_2_ capnography as a potential predictor for OHCA outcome.

Our findings may have important clinical implications. The results of this analysis highlight that there are many dimensions of EtCO_2_ that may better guide resuscitation. Using the change in capnography throughout resuscitation may be an advancement over using discrete EtCO_2_ cut-offs,^14^ although our findings require further validation. Additional questions that remain include the duration of EtCO_2_ capnography monitoring necessary to determine a reliable temporal change estimate. Naturally, this is not the only way to analyze these data. Other potential approaches include machine learning algorithms or inclusion of peak volume or thoracic compliance. These are complimentary targets for future projects. Validation of this approach (EtCO_2_ trend monitoring) can be useful in resuscitation and merits independent validation prior to clinical application.

### Limitations

This study has limitations. These data are a retrospective review of one-third of previously collected data from agencies involved in a clinical trial performed more than 7 years ago. Generalizability of ventilation quality metrics from potentially high-performing emergency medical services agencies may not be broadly applicable.^26,27^ Furthermore, the clinical trial evaluated the effectiveness of airway device on OHCA outcomes. We attempted to adjust for interventions such as airway choice, ventilation quality, chest compression quality, and epinephrine given. However, we are unable to adjust for defibrillation timing, cumulative dosing of epinephrine or other medications such as sodium bicarbonate as it is not available or underpowered in this initial dataset. We also are unable to evaluate newer measurable ventilation metrics such as tidal volume. Additionally, we evaluated 1 characteristic of EtCO_2_ over resuscitation. Other continuous capnography quality metrics such as airway opening index may be contributing to OHCA outcomes as well.^28^

## Conclusions

This secondary analysis found that dynamic changes in EtCO_2_ were associated with OHCA outcomes. These data suggest value in using continuous waveform capnography in resuscitation.

## Notes

Supplement 1.
Trial Protocol

Supplement 2. **eAppendix 1.** Schematic of Time Points **eAppendix 2.** Variability in Capnography Change **eAppendix 3.** Stratified Analysis by Resuscitation Length of Time **eAppendix 4.** Generalized Estimated Equations to Account for Trial Design

Supplement 3. Data Sharing Statement
