# Supplementary material for: Temporal Trends in End-Tidal Capnography and Outcomes in Out-of-Hospital Cardiac Arrest: A Secondary Analysis of a Randomized Clinical Trial
Source: JAMA Netw Open. 2024 Jul 5;7(7):e2419274. doi: 10.1001/jamanetworkopen.2024.19274 (PMC11227078; doi:10.1001/jamanetworkopen.2024.19274)

Pragmatic Trial of Airway Management in Out-of-Hospital Cardiac Arrest

(Pragmatic Airway Resuscitation Trial)

Clinical Trial Protocol

Version 5.0 February 19, 2015 –revised and approved protocol

Version 5.1 April 1, 2015 –Minor changes to DSMB revised and approved protocol

Version 6.0 July 13, 2016 – Minor updates

Version 7.0 November 29, 2016 – Revisions

PRAGMATIC AIRWAY RESUSCITATION TRIAL

ROC

***This trial protocol is adapted with permission from the ROC CCC Trial protocol.
This protocol may not be used or reproduced without permission.***

TABLE OF CONTENTS

1. Key Study Information 5
1.1 Clinical Trial Registration 5
1.2 Funding and Support 5
1.3 Study Leadership and Key Personnel 5
1.3.1 Principal Investigator, Lead Facilitator 5
1.3.2 Co-Investigators 5
2. Acronyms and Abbreviations 6
3. SUMMARY - Pragmatic Trial of Airway Management in Out-of-Hospital Cardiac Arrest (Pragmatic Airway Resuscitation Trial) 8
4. Study Objective 11
5. Background 11
5.1 Conceptual Framework 11
6. Pertinent Existing Evidence on Endotracheal and Laryngeal Tubes 14
6.1 Animal 14
6.2 Human 14
6.2.1 Observational Studies 14
6.2.2 Randomized Controlled Trials 16
7. Existing EMS Airway Practices 16
8. Overview of Trial Design 17
9. Inclusion Criteria 17
10. Exclusion Criteria 17
11. Trial Setting 18
12. Interventions 19
12.1 EMS Personnel Training Levels 19
12.2 Primary Endotracheal Intubation (Control Arm) 19
12.2.1 ETI Device and Technique 19
12.2.2 ETI Techniques Permitted in the Trial 20
12.3 Primary Laryngeal Tube Insertion (Intervention Arm) 20
12.3.1 LT Device and Technique 20
12.3.2 Limitation of the Trial Intervention Arm to the Laryngeal Tube 21
12.4 Research Procedures 21
12.5 Protocol Considerations 22
12.5.1 Number of Airway Insertion Attempts 22
12.5.2 Unsuccessful Airway Insertion and Rescue Interventions 23
12.5.3 Confirmation of Airway Placement 23
12.5.4 Use of Neuromuscular Blocking Agents 24
12.5.5 Absence of a Bag-Valve-Mask-Only Arm 24
12.5.6 Emergency Department Management of the EMS Airway Device 24
12.5.7 EMS Personnel Airway Management Experience 25
12.5.8 Post-Arrest Care 25
13. Random Allocation and Blinding 26
13.1 Random Allocation Method 26

13.2 Rationale for Cluster Randomization 27
13.3 Blinding of Treatment Assignment 27

14. Outcomes 27
14.1 Primary Outcome 27
14.1.1 72-Hour Survival 27
14.2 Secondary Outcomes 28
14.2.1 Return of Spontaneous Circulation 28
14.2.2 Airway Management Course 28
14.2.3 Survival to Hospital Discharge 29
14.2.4 Neurologically-Intact Survival to Hospital Discharge 29
14.3 Adverse Events 29
14.3.1 Unexpected Adverse Events (UAE) 30
14.3.2 Expected Adverse Events 30
14.4 Other Data 31
14.4.1 CPR Process Data 31
14.4.2 Other Explanatory Mechanisms 31

15. Analysis Plan 32
15.1 CONSORT Diagram 32
15.2 Intention-to-Treat vs As-Treated 32
15.3 Primary Analysis – 72-Hour Survival 32
15.4 Secondary Analyses 33
15.5 Subgroup Analyses 33

16. Sample Size 33
16.1 Sample Size Estimate 33
16.2 Interim Monitoring Plan and Stopping Boundaries – Criteria for Terminating the Trial 34

17. Eligibility for Participating and Remaining in the Enrollment Phase of the Trial 35

18. Timeline 35
18.1 Milestones for the Planning Phase 35
18.2 Milestones for Trial Execution and Subject Recruitment 36

19. Data Sources and Management 36
19.1 Data Sources 36
19.1.1 Out-of-Hospital EMS Data 36
19.1.2 Hospital Data 36
19.1.3 Post-Discharge Vital Status 37
19.2 Data Entry 37
19.3 Database Management 37

20. EMS Research Protocol Training 37
20.1 Overview of EMS Protocol Training Elements 37
20.2 Retraining and Feedback 38

21. Guidance for Receiving Emergency Departments 38

22. Study Monitoring 39
22.1 Study Monitoring Committee 39
22.2 Data Safety and Monitoring Board 39

23. Regulatory Considerations 40
23.1 Human Subjects 40

23.2 Food and Drug Administration 40
23.2.1 Technical and Regulatory Information for the ET Tube 40
23.2.2 Technical and Regulatory Information for the LT 41

24. References 42

25. Appendix 1 – Summary of Protocol Amendments 51
25.1 Version 4.2, December 10, 2014 51
25.2 Version 5.0, February 19, 2015 51
25.3 Version 5.1, April 1, 2015 51
25.4 Version 6.0, June 27, 2016 51

26. Appendix 2 – List of Study Personnel 52
26.1 Principal Investigator, Lead Facilitator 52
26.2 Co-Investigators 52
26.3 Liaisons with the Resuscitation Outcomes Consortium 52
26.4 Project Direction and Coordination 53
26.5 Biostatisticians 53
26.6 Liaisons with the National Heart, Lung and Blood Institute 54

27. Appendix 3 – Criteria to Enter and Remain in the Enrollment Phase of the Trial 55
27.1 Criteria to Enter Enrollment Phase of Trial 55
27.2 Criteria to Remain in Enrollment Phase of Trial 55

28. Appendix 4 – Additional Information Justifying the Use of Exception from Informed Consent (EFIC) for Emergency Research 56

29. Appendix 5 – Example Patient Notification Forms for Use Under Exception from Informed Consent for Emergency Research 61
29.1 Notification Forms (for initially surviving Subjects) 61
29.2 Next of Kin Notification Letter (for family of subjects who did not survive) 64
29.3 Study Information Sheet (accompanies Next of Kin Notification Letter) 66
29.4 Script for EMS Notification of Next of Kin 67
29.5 Sample Community Consultation Plan 68

1. Key Study Information

1.1 Clinical Trial Registration

This study is registered with www.clinicaltrials.gov as trial no. NCT02419573.

1.2 Funding and Support

The planning phase of this study is supported by grant award UH2-HL125163 from the National Heart, Lung and Blood Institute (NHLBI).

Additionally, the ROC data coordinating center and sites are supported through a series of cooperative agreements through the NHLBI (5U01 HL077863-University of Washington Data Coordinating Center, HL077866-Medical College of Wisconsin, HL077867University of Washington, HL077871-University of Pittsburgh, HL077873-Oregon Health and Science University, HL077881-University of Alabama at Birmingham, HL077887-University of Texas SW Medical Center/Dallas, HL077908-University of California San Diego).

The trial execution phase of this study is supported by grant award UH3-HL125163 from NHLBI.

1.3 Study Leadership and Key Personnel

This study is led by investigators and research personnel from the Department of Emergency Medicine, University of Alabama School of Medicine, Birmingham, Alabama and the Clinical Trials Center, University of Washington, Seattle, Washington. Execution of research activities will be facilitated by US regional coordinating centers of the Resuscitation Outcomes Consortium.

1.3.1 Principal Investigator, Lead Facilitator

Henry E. Wang, MD, MS
Professor and Vice Chair for Research
Department of Emergency Medicine
University of Alabama School of Medicine
(205)-996-6526/(205)-410-1267
hwang@uabmc.edu

1.3.2 Co-Investigators

Susanne May, PhD
Professor
Principal Investigator
ROC Clinical Trial Center
University of Washington
206-685-1302
sjmay@uw.edu

Graham Nichol, MD
Professor
Medical Director, ROC Clinical Trial Center
University of Washington
206-685-1302
nichol@uw.edu

2. Acronyms and Abbreviations

ACLS	Advanced Cardiac Life Support
AED	Automated External Defibrillator
ALPS	Amiodarone, Lidocaine or Placebo Study
ALS	Advanced Life Support
BLS	Basic Life Support
CARES	Cardiac Arrest Registry to Enhance Survival
CC/PD	Community Consultation/Public Disclosure
CCC	Continuous Chest Compression
CI	Confidence Interval
CONSORT	CONsolidated Standards Of Reporting Trials
CPR	Cardiopulmonary Resuscitation
CTC	Clinical Trial Center (University of Washington)
DCC	Data Coordinating Center
DNAR	Do Not Attempt Resuscitation
DSMB	Data Safety and Monitoring Board
ECG	Electrocardiogram
ED	Emergency Department
EFIC	Exception From Informed Consent
EMS	Emergency Medical Services
EMT	Emergency Medical Technician
EOA	Esophageal Obturator Airway
ET	Endotracheal
ETC	Esophageal Tracheal Combitube
ETI	Endotracheal Intubation
FDA	Food and Drug Administration
ICU	Intensive Care Unit

IDE	Investigational Drug Exemption
IRB	Institutional Review Board
KLT	King Laryngeal Tube
LT	Laryngeal Tube
LTAC	Long Term Acute Care Facility
LVAD	Left Ventricular Assist Device
MRS	Modified Rankin Score
NHLBI	National Heart, Lung, and Blood Institute
NIH	National Institutes of Health
NMB	Neuromuscular Blocking/Blockade
NRB	Non-Rebreather Mask
OHCA	Out-of-Hospital Cardiac Arrest
PART	Pragmatic Airway Resuscitation Trial
PCI	Percutaneous Coronary Intervention
PI	Principal Investigator
PRIMED	Prehospital Resuscitation IMpedance valve and Early vs Delayed analysis
ROC	Resuscitation Outcomes Consortium
ROSC	Return of Spontaneous Circulation
RSI	Rapid Sequence Intubation
SGA	Supraglottic Airway
SMC	Study Monitoring Committee
SUSAR	Suspected Unexpected Serious Adverse Reaction
TAH	Total Artificial Heart
TH	Therapeutic Hypothermia
TTM	Targeted Temperature Management
UAB	University of Alabama
US	United States
UW	University of Washington

3. SUMMARY - Pragmatic Trial of Airway Management in Out-of-Hospital Cardiac Arrest (Pragmatic Airway Resuscitation Trial)

Aim: The objective of this pragmatic trial is to compare the effectiveness of primary (initial) endotracheal intubation (ETI) versus primary (initial) laryngeal tube (LT) airway management strategies upon 72-hour survival after out-of-hospital cardiac arrest (OHCA).

Rationale: Cardiopulmonary arrest is the sudden cessation of heart function and circulatory blood flow resulting in whole-body ischemia. Sudden out-of-hospital cardiopulmonary arrest (OHCA) is a major public health problem affecting over 300,000 adults in the United States each year, with only 1 in 10 of treated patients surviving.[1] Successful OHCA resuscitation requires circulation of oxygen to injured organs such as the heart and brain. Airway management is the process of opening the mouth and throat to deliver oxygen to the lungs for circulation to the vital organs.

Endotracheal intubation (ETI), the insertion of a plastic breathing tube through the mouth and into the trachea, is the most common advanced airway management technique in OHCA resuscitation in North America. As the first to provide critical care in the out-of-hospital setting, paramedics in North America have performed ETI on OHCA victims for over 30 years. Despite its routine use, ETI involves many pitfalls. Adverse events associated with ETI include unrecognized tube misplacement or dislodgement, repeated intubation attempts, iatrogenic hyperventilation, hypoxia and bradycardia, and unintended interruptions in cardiopulmonary resuscitation (CPR) chest compression continuity.[2-7] In the US, resources for paramedic ETI training are often inadequate.[8] Individual clinical experience is also sparse; in Pennsylvania, paramedics perform a median of one (1) ETI annually.[9]

Supraglottic airways (SGA) such as the Laryngeal Tube (King Systems, Noblesville, IN), Combitube (Covidien, Ltd, Mansfield, MA), Laryngeal Mask Airway (LMA North American, San Diego, CA) and i-Gel (Intersurgical, Inc., Liverpool, NY) are simpler than ETI yet ventilate as well. Because of the fewer pitfalls and training requirements, many paramedics favor primary SGA over ETI. However, it is unclear if outcomes are improved with primary SGA- versus ETI-based airway management strategies. The LT is the most common SGA used by EMS personnel in the United States.[10]

Hypotheses: The primary null hypothesis is that 72-hour survival is similar between primary Laryngeal Tube (LT) SGA and primary ETI airway management strategies. Evaluated secondary outcomes will include return of spontaneous circulation, survival to hospital discharge, neurologically intact survival at hospital discharge, airway management performance, and clinical adverse events.

Study Design: This trial will use a cluster randomized design with periodic crossover. Responding EMS personnel will perform airway management using either a strategy of primary ETI (control) or primary LT insertion (intervention). The trial will be designed to detect the superiority of either primary ETI or primary LT airway management over the other intervention arm.

Study Population:

- Inclusion Criteria: Adult (age ≥18 years or per local interpretation), non-traumatic OHCA with EMS personnel on scene and requiring advanced airway insertion (ETI, LT) or ventilatory support such as by bag-valve-mask device or non-rebreather mask.

- Exclusion Criteria: Known pregnant women, known prisoners, patients with major facial trauma, major bleeding or exsanguination, patients receiving initial care by non-PART participating EMS agency capable of performing ETI, LT or other advanced airway insertion, patients with ET tube, LT or other advanced airway insertion prior to ROC EMS arrival, patients with a pre-existing tracheostomy, obvious asphyxial cardiac arrest (choking or hanging), patients with a left ventricular assist device (LVAD) or total artificial heart (TAH), patients with pre-existing "do-not-attempt-resuscitation" (DNAR) orders, inter-facility transports, and patients with a "do not enroll" bracelet.

Study Therapies:

- **1) Primary Endotracheal Intubation** (Control): Advanced-level EMS personnel will use ETI as the primary (initial) advanced airway management intervention. Basic-level EMS personnel will use bag-valve-mask (BVM) ventilation only.

- **2) Primary Laryngeal Tube Supraglottic Airway Insertion** (Intervention): Advanced-level EMS personnel will use the Laryngeal Tube (LT) as the primary (initial) advanced airway management intervention, and basic-level EMS personnel will use bag-valve-mask ventilation. If trained to use LT, basic-level EMS personnel may perform LT insertion as the primary (initial) airway intervention.

EMS agencies that do not utilize LT will be excluded from the trial. All other aspects of resuscitation will follow local EMS protocols, including chest compression methods, timing of rhythm analysis, and insertion of intravenous lines and administration of medications. If allowed by local protocol, advanced and basic-level EMS personnel may use a non-rebreather (NRB) mask instead of BVM ventilation.

Outcomes:

The primary outcome will be:

1) **72-hour hospital survival**, defined as patient status (alive/dead) at 72 hours after the initial onset of cardiac arrest.

Secondary outcomes will include:

1) **Return of Spontaneous Circulation (ROSC)**, defined as the presence of palpable pulses upon ED arrival.

2) **Airway Management Clinical Course,** including sequence of ETI/LT insertion, ETI/LT insertion success and time, number of ETI/LT insertion attempts, and airway conversion (change of LT-to-ETI, or ETI-to-LT) in the receiving Emergency Department.

3) **Adverse Events,** including initially unrecognized airway misplacement (including endotracheal tube misplacement), inadequate ventilation requiring airway exchange, vomiting, oropharyngeal, hypopharyngeal or gastrointestinal trauma, and pneumothorax.[4,11]

4) **Hospital Survival,** defined as patient status (alive/dead) at hospital discharge.

5) **Hospital Survival with Good Neurologic Function,** defined as Modified Rankin Score ≤3 upon hospital discharge.

Analysis: We will calculate the difference in outcomes divided by the estimated "robust" standard error based on the Huber-White sandwich estimator in order to account for within cluster correlation and variability, which might depart from the classical assumptions.[12] To quantify the treatment effect, we will calculate the 95% CI for the difference in event rates. We will perform the analysis on an intention-to-treat (primary analysis) as well as treatment-received bases (secondary analysis).

Sample Size: The trial will require 2,612 subjects (1,306 per group) to have 85% power to detect a 4.5% absolute difference in 72-hour survival, given baseline survival of 13.7% and an overall two-sided alpha of 0.05. To allow for subject withdrawal and lost-to-follow-up, the trial will enroll up to 3,000 subjects.

Regulatory and Ethical Considerations: The trial will be carried out under federal rules for Exception from Informed Consent (EFIC) outlined by the guidance document "Waiver of Informed Consent Requirements in Certain Emergency Research" (Federal Register, Vol. 61, pp. 51531-51533, November 1, 1996 - http://www.gpo.gov/fdsys/pkg/FR-1996-10-02/html/96-24968.htm), OHRP "Basic HHS Policy for Protection of Human Research Subjects" (45 CFR 46.101(i) - http://www.hhs.gov/ohrp/humansubjects/guidance/45cfr46.html#46.101), and FDA regulations 21 CFR 50.24.

4. Study Objective

The objective of this pragmatic trial is to compare the effectiveness of primary ETI versus primary LT airway management strategies upon 72-hour survival after OHCA.

5. Background

5.1 Conceptual Framework

Out-of-Hospital Cardiac Arrest is a Major Public Health Problem. Cardiopulmonary arrest is the critical illness of whole-body ischemia resulting from sudden cessation of heart function and circulatory blood flow. Sudden out-of-hospital cardiopulmonary arrest (OHCA) is a major public health problem affecting over 300,000 adults in the United States each year.[1] Despite organized international efforts, only about 1 in 10 survive after OHCA. New strategies are needed to improve rates of neurologically intact survival from this devastating condition.

Airway Management is Important in Cardiac Arrest Treatment. Successful resuscitation from OHCA requires delivery of life-saving oxygen to injured organs such as the heart and brain. Airway management is the process of opening the mouth and throat to deliver oxygen to the lungs. In the cardiac arrest victim, the oxygen delivered to the lungs is subsequently circulated to the vital organs through cardiopulmonary resuscitation (CPR) chest compressions.

Endotracheal Intubation is the Most Common Airway Management Technique. Basic techniques for airway management include "head-tilt/chin lift" and the jaw thrust. However, to optimize airway patency, rescuers usually opt for more advanced airway management techniques. In North America, endotracheal intubation (ETI) is the most common advanced airway management technique in both in-hospital and out-of-hospital settings. (Figure 1) ETI (or "intubation") involves exposure of the vocal cords using a large lighted metal laryngoscope blade inserted in the mouth and insertion of a plastic breathing tube through the throat, between the vocal cords and into the trachea. ETI is the preferred method for airway management because it provides a direct conduit to the lungs, facilitates easier and more controlled delivery of oxygen, and protects the lungs from aspiration of vomitus. ETI is a complex technique involving over 100 psychomotor steps and is usually performed only by physicians or specially trained healthcare providers.[13]

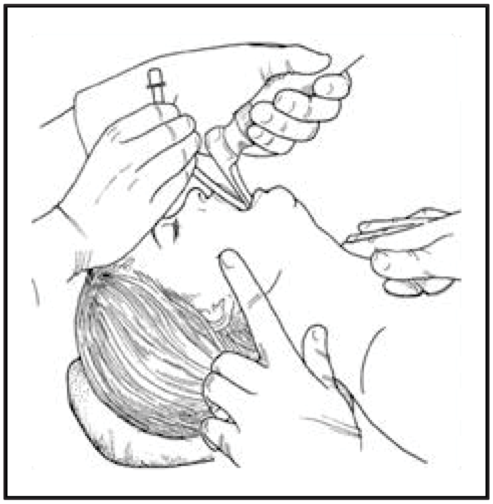

Figure 1 - Endotracheal Intubation

Intubation is Standard of Paramedic Cardiac Arrest Care But Has Many Pitfalls. As the initial provider of critical care in the field, US paramedics have performed ETI on OHCA victims for over 30 years.[14-17] However, numerous studies highlight the pitfalls associated with paramedic ETI. For example, unrecognized misplacement of the endotracheal tube (in the esophagus rather than the trachea) has been reported in up to one-fourth of paramedic ETI.[18] While the endotracheal tube should ideally be placed on the first attempt, in a large series of 1,271 OHCA, we found that one-fourth of ETI efforts required two or more attempts.[6] While select studies report paramedic ETI

success rates >90%, using US national data we observed ETI success rates of only 70%.[19] Paramedic ETI may lead to subsequent iatrogenic hyperventilation, which decreases coronary blood flow during CPR.[20-22]

ETI may result in CPR Interruptions. Interruptions in CPR chest compressions – including even brief 20-second interruptions – may disrupt coronary blood flow, impairing OHCA survival.[23,24] Using state-of-the-art cardiac monitor technology, we were able to identify chest compression interruptions associated with intubation efforts by City of Pittsburgh paramedics.[7] In this series of 100 OHCA, ETI efforts resulted in a median of two (2) CPR interruptions (IQR 1-3; range 1-6), totaling a median of 109.5 seconds (IQR: 54-198; range 13-446). One-fourth of the CPR interruptions exceeded three (3) minutes. (Figure 2)

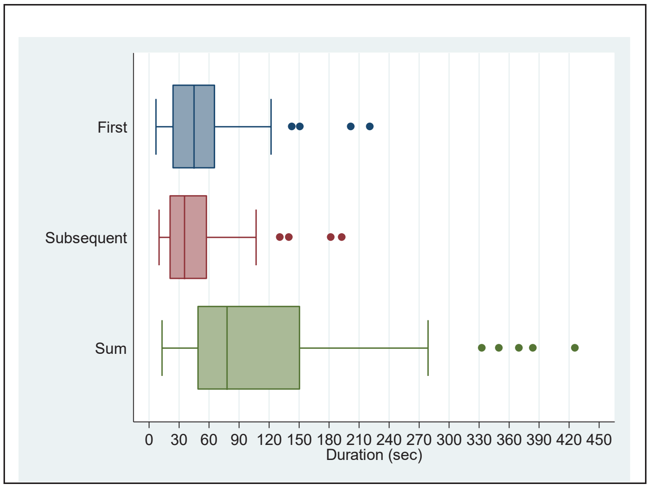

Figure 2 – Duration of CPR chest compression interruptions from paramedic intubation efforts.

Paramedic Intubation Training and Experience are Inadequate. While ETI is complex, opportunities for baseline training and continuing experience are inadequate.[9,25] Compared with the 35-200 ETI required for emergency medicine residents, anesthesiology residents and nurse anesthetist trainees, US paramedic students must perform only five (5) ETI prior to graduation.[26-32] While the customary setting for learning ETI is in the operating room training under the tutelage of anesthesiologists, students at most nationally accredited paramedic training programs receive only 2-4 days of this training experience.[8] Some training programs are not able to provide any operating room training. Mannequins do not adequately recreate the "feel" of live human airways.

Regular clinical experience is important for maintaining ETI skill. Throughout Pennsylvania in 2003, practicing paramedics performed a median of one (1) ETI (interquartile range 0-3) annually; 39% performed no ETI, and 67% performed fewer than two (2) ETI.[33] In Maine, Burton, et al. found that only 40% of paramedics attempted ETI annually, and only 1-2% of all paramedics performed five (5) or more ETI annually.[34]

Supraglottic Airways (SGA) - Simpler Alternatives to Intubation. Supraglottic airways (SGA) offer simpler alternatives to ETI. SGA in current North American EMS use include the Laryngeal Mask Airway (LMA), Combitube and King Laryngeal Tube.[35,36] SGA are inserted "blindly"

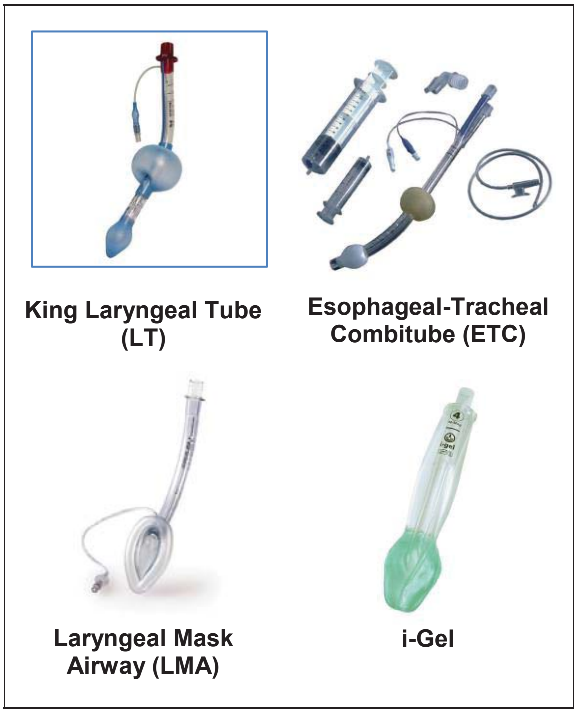

Figure 3 – Supraglottic Airways (SGA)
Only the King Laryngeal Tube (LT) will be tested in this study.

through the mouth and sit outside of the vocal cords.[37] (Figure 3) Compared with ETI, SGAs offer simple and expeditious operation, lower skill acquisition and maintenance thresholds, and similar ventilatory characteristics. While previously reserved for contingency use in the event of unsuccessful ETI efforts, many EMS agencies now perform advanced airway management using a strategy of primary SGA insertion.[38] While there are no relevant national data available, we are aware of this practice in select communities, including Kalamazoo, Michigan; Chesterfield County, Virginia; Collier County, Florida; and Dallas, Texas.

Observational Data Offer Unclear Guidance. SGA are more pragmatic than ETI and would be expected to result in similar or better outcomes after OHCA However, OHCA outcomes after SGA insertion are unclear. In a secondary analysis of data from the Resuscitation Outcomes Consortium PRIMED study, we studied 10,455 adult OHCA, including 8,487 (81.2%) who received ETI and 1,968 (18.8%) who received SGA.[10] Compared with SGA, ETI was independently associated with higher adjusted odds of return of spontaneous circulation, survival to discharge, and survival to discharge with good neurologic function, even after multivariable adjustment for confounders. (Figure 4)

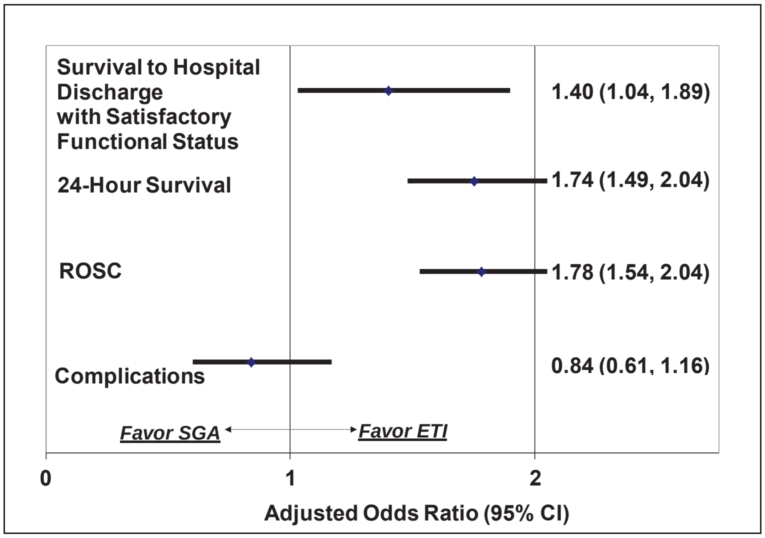

Figure 4 - Preliminary Data – OHCA outcomes for ETI vs. SGA. Data from secondary retrospective analysis of 10,455 OHCA from the ROC PRIMED trial.[10]

We performed a similar analysis using 8,701 adult OHCA from the national Cardiac Arrest Registry to Enhance Survival (CARES), including 5,591 (64.3%) ETI and 3,110 (35.7%) SGA.[39] We found similar results associating ETI with better outcomes than SGA, even after multivariable and propensity score adjustment and matching. Other observational studies have reported similar associations.[40-43]

Need for a Randomized Trial. Important analytic limitations of prior observational airway studies include a) the influence of confounding-by-indication, and b) the absence of information on practitioner skill, device preference, EMS agency protocols, and perception of patient severity.[10,39-42,44] These uncertain results from observational studies highlight that a randomized trial is the best way to differentiate outcomes between airway management strategies involving primary ETI and primary SGA insertion.

To date, the only clinical trials of out-of-hospital airway management have not focused on adult OHCA or the LT. Gauche, et al. randomized children to different paramedic airway studies but included a heterogeneous disease population.[45] Bernard, et al. randomized intubation techniques but focused on traumatic brain injury.[46] Frascone, et al. randomized out-of-hospital patients to ETI or LT, but the study focused on airway placement success rates, not patient outcomes.[47] A recent pilot trial compared ETI with SGA use in adult OHCA, but the study occurred in the United Kingdom, which has different EMS protocols, practices and airway device preferences than in the US.[48]

6. Pertinent Existing Evidence on Endotracheal and Laryngeal Tubes

6.1 Animal

There have been only limited evaluations of LT outcomes in animal models of cardiac arrest. The feasibility of LT use has been demonstrated in porcine and rabbit models.[49,50]

Segal, et al. compared the effect of the ET tube, LT, Combitube and laryngeal mask airway (LMA) upon carotid blood flow in a porcine model of cardiac arrest.[51] In this study, carotid blood flow was determined by Doppler imaging, with post-mortem arteriograms in select animals. The authors found that carotid blood flow was lower for all supraglottic airway devices than ETT; [King (median 10 ml/min; IQR 6-41), LMA (10; 4-39), and Combitube (5; -0.4-15)] versus ETT (21; 14-46) ($p<0.05$ for each SGA compared with ETT). The study was not powered to evaluate ROSC or survival endpoints.

6.2 Human

6.2.1 Observational Studies

Several analyses of adult OHCA cohorts have compared outcomes between patients receiving ETI and those receiving other airway management techniques, including SGA devices.

Fouche, et al. performed a meta-analysis comparing differences in survival between advanced airway devices and basic airway interventions.[52,53] Compared with basic airway interventions, ETI and SGA insertion were associated with poorer longer-term outcomes. However, the meta-analysis did not directly compare outcomes between ETI and SGA.

Select observational studies have directly compared outcomes between adults OHCA receiving ETI and those SGA (including the LT). (Table 1) Of note, there have been no direct comparisons between ETI and LT. Furthermore, these studies have important limitations, including retrospective designs, limited data on airway management devices used or the course of care, incomplete risk adjustment and confounding by indication. A prospective study with random allocation is necessary to overcome the limitations of these observational studies.

Study	Population	Comparison	Primary Findings	Limitations
Wang, et al., Resuscitation 2013[10]	ROC Network N=10,455<br><br>(8,487 ETI, 1,968 SGA)	ETI vs. SGA (LT, LMA, Combitube)	ETI associated with increased survival to hospital discharge (adjusted OR 1.40; 95% CI: 1.04, 1.89), ROSC (1.78; 1.54, 2.04) and 24-h survival (1.74; 1.49, 2.04) than SGA.	Did not segregate SGA subtypes<br><br>Did not evaluate BVM-only<br><br>Confounding by indication
McMullan, et al., Resuscitation, 2014[54]	CARES Network n=10,691<br><br>(5,591 ETI, 3,110 SGA, 1,929 none/BVM)	ETI vs. SGA (LT, LMA, Combitube)<br><br>ETI vs. BVM	ETI associated with higher sustained ROSC (adjusted OR 1.35; 95% CI 1.19-1.54), survival to hospital admission (1.36; 1.19-1.55), hospital survival (1.41; 1.14-1.76) and hospital discharge with good neurologic outcome (1.44; 1.10-1.88) than SGA.<br><br>BVM associated with higher survival to hospital admission (1.31; 1.16-1.49), hospital survival (2.96; 2.50-3.51) and hospital discharge with good neurologic outcome (4.24; 3.46-5.20) than [ETI or SGA].	Did not segregate SGA subtypes<br><br>Confounding by indication
Hasegawa, et al., JAMA 2013[41]	All-Japan Utstein N=649,359<br><br>(41,972 ETI, 239,550 SGA, 367,837 BVM)	ETI vs. SGA vs. BVM	One-month neurologically favorable survival lower for ETI (adjusted OR 0.41; 95% CI, 0.37-0.45) and SGA (0.38; 0.36-0.40) than BVM.	LMA most common SGA<br><br>Limited experience with ETI.<br><br>No direct comparison of ETI vs. SGA<br><br>Confounding by indication
Tanabe, et al. J Emerg Med 2013[43]	All-Japan Utstein N=138,248<br><br>(16,054 ETI, 34,125 LMA, 88,069 Esophageal-Obturator Airway [EOA])	ETI vs. LMA vs. EOA	One-month neurologically favorable lower for LMA (aOR 0.77; 95% CI: 0.64-0.94) and EOA (0.81; 0.68-0.96) than ETI.	Limited experience with ETI<br><br>Many EOA<br><br>Confounding by indication
Shin, et al., Resuscitation 2012[55]	Korean national OHCA database N=5,278<br><br>(250 ETI, 391 LMA, 4,637 BVM)	ETI vs LMA vs. BVM	Survival to hospital discharge similar for ETI vs BVM (aOR 1.00; 0.60-1.66) but lower for LMA vs. BVM (0.52; 0.32-0.85).	Very few ETI or LMA<br><br>Confounding by indication

Table 1 – Observational studies comparing ETI with SGA in adult OHCA.

6.2.2 Randomized Controlled Trials

There have been few out-of-hospital randomized controlled studies of ETI or the LT. There have been no randomized trials comparing the effect of ETI and LT upon patient outcomes in adult OHCA.

Gausche, et al. conducted one of the few randomized controlled trials of out-of-hospital ETI.[45] In this study of 830 children requiring airway management, paramedics altered treatment between BVM (control) or BVM+ETI (intervention). Randomization occurred in odd/even day format. There was no significant difference in survival between the BVM group (123/404 [30%]) and the ETI group (110/416 [26%]) (OR 0.82; 95% CI: 0.61-1.11) or in the rate of achieving a good neurological outcome (BVM, 92/404 [23%] vs ETI, 85/416 [20%]) (OR 0.87; 95% CI: 0.62-1.22).

Frascone, et al. compared ETI and LT performance in a randomized series of 213 adults requiring airway management.[47] Randomization was performed at the patient level using blinded airway management pouches. The overall placement success rate was virtually equal across the two groups (ETI=80.2%, King LT=80.5%; p=0.97). The median time to placement between ETI and the King LT was also not significantly different (ETI=19.5 seconds vs. King LTS-D=20.0 seconds; p=0.80). The study was strictly focused on airway insertion performance and did not evaluate patient outcomes.

Benger, et al. have completed the pilot phase of a trial in the United Kingdom comparing ETI versus i-Gel and LMA insertion in adult OHCA.[48] Randomization occurred by participating paramedic. From a total of 1,375 OHCA undergoing active resuscitation, 615 patients were enrolled (232 i-Gel, 174 LMA, 209 usual practice [ETI]). The LMA arm was suspended after 10 months due to staff safety concerns (forceful expulsion of blood and/or stomach contents from the gastric port of the LMA).[56] The rates of transport to hospital, return of spontaneous circulation and hospital admission did not differ (p=0.28, p=0.91 and p=0.58 respectively). Survival to discharge was: i-Gel 10.3%, LMA 8.0%, usual practice 9.1% (p=0.73) and to 90 days was: i-Gel 9.5%, LMA 6.9%, usual practice 8.6% (p=0.65). Neurocognitive function and quality of life at 90 days were not significantly different. The study group will soon start a larger scale trial of n=9,000 adult OHCA to more definitively ascertain outcome differences between ETI and i-Gel.

7. Existing EMS Airway Practices

Historically, US paramedics have favored ETI over SGA for airway management of adult OHCA. However, a shift in practice occurred in the US approximately 5 years ago, with multiple US EMS agencies switching to SGA as the primary device in OHCA resuscitation. Several ROC EMS agencies (including those affiliated with the Dallas, Pittsburgh, and Portland centers) currently utilize SGA as the primary airway management device in patients with OHCA. (Table 2)

Supraglottic Airway Used by EMS Agency	
King Laryngeal Tube	2[illegible]
Combitube	[illegible]
Laryngeal Mask Airway	[illegible]
i-Gel	[illegible]
Supraglottic Airway Used by	
Primary Airway Used in Cardiac Arrest	
Endotracheal Intubation	2[illegible]
Supraglottic Airway (paramedic discretion)	[illegible]
Supraglottic Airway (paramedic protocol)	[illegible]
Other	[illegible]

Table 2 - Airway management practices of 33 US EMS Agencies in the Resuscitation Outcomes Consortium

We have verified in extensive discussions with the ROC EMS Operations committee that paramedics are willing to randomize OHCA patients to ETI or LT.

8. Overview of Trial Design

This trial will use a cluster randomized controlled design with periodic crossover. Responding EMS personnel will perform advanced airway management using either primary ETI (control) or primary LT insertion (intervention). The trial will be designed to detect the superiority of either primary ETI or primary LT airway management over the other intervention arm.

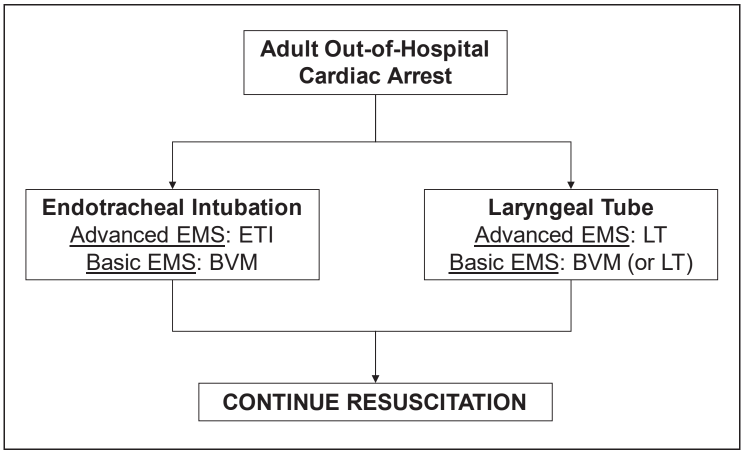

Figure 5 - Overview of Trial Protocol
ETI = Endotracheal Intubation; SGA = Supraglottic Airway
BVM = Bag-Valve-Mask Ventilation

9. Inclusion Criteria

This trial will include subjects meeting all of the following conditions:

a) Out-of-hospital cardiac arrest (OHCA);

b) Adult (age ≥18 years or per local interpretation);

c) Non-traumatic etiology;

d) Initiation of ventilator support (e.g. Bag-valve-mask device or non-rebreather mask.

The trial will include EMS-witnessed arrests. The trial will include individuals experiencing recurrent OHCA during the same care episode.

If a patient receives bag-valve-mask ventilation but not ETI or SGA, the patient will be included in the study per intention-to-treat principles, regardless of whether the patient regains consciousness or remains comatose. If allowed by local protocol, EMS personnel may use a non-rebreather (NRB) mask instead of BVM ventilation; these patients will similarly be included in the study per intention-to-treat principles.

10. Exclusion Criteria

This study will exclude the following subjects:

Protected Populations

a) Known pregnant women;

b) Known prisoners;

Trauma

c) Major facial trauma (visible major deformity, copious oral bleeding, etc);

d) Major bleeding or exsanguination (e.g., major upper or lower GI bleed, visceral perforation, major uncontrolled bleeding from laceration or injury);

Pre-Existing Conditions or Prior Treatment

e) Patient receiving initial care by a non-PART participating EMS agency capable of performing ETI, LT or other advanced airway management.

f) Patients with ET tube, LT or other advanced airway device inserted prior to participating EMS agency arrival (e.g., inserted by healthcare facility personnel);

g) Patients with a pre-existing tracheostomy;

h) Obvious asphyxial cardiac arrest (e.g., choking, foreign body aspiration, angioedema, epiglottitis, trauma to mouth and face, etc.);

i) Patients with a left ventricular assist device (LVAD) or total artificial heart (TAH);

j) Patients with pre-existing written "do-not-attempt-resuscitation" (DNAR) orders;

k) Inter-facility transports;

Other Exclusions

l) Patients with a "do not enroll" bracelet.

11. Trial Setting

EMS agencies associated with US Regional Coordinating Centers of the Resuscitation Outcomes Consortium will be selected for participation in this trial. (Figure 6) Conditions for selection include:

a) US EMS agency;

b) Affiliation with US ROC Regional Coordinating Centers;

c) Use of the King LT as the primary SGA;

EMS agencies that do not utilize King LT SGA will be excluded from the trial.

Per the requirements of the NIH/NHLBI award supporting this clinical trial (RFA-HL-14-019, http://grants.nih.gov/grants/guide/rfa-files/RFA-HL-

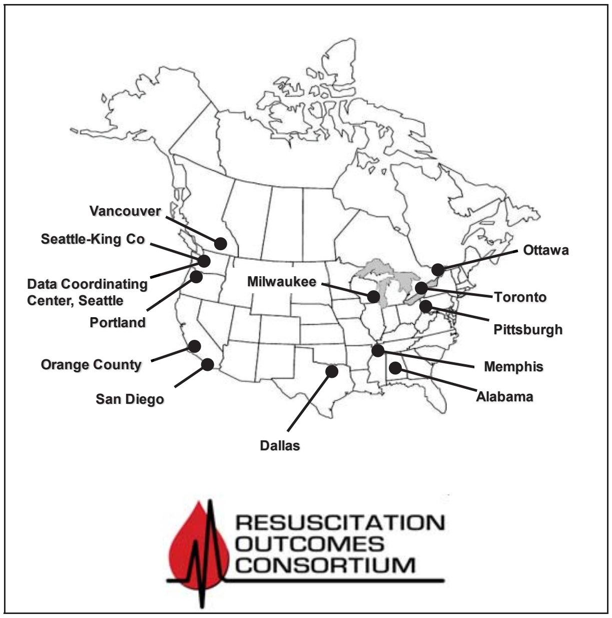

Figure 6
The Resuscitation Outcomes Consortium
Only select US sites (Alabama, Dallas, Milwaukee, Oregon, Pittsburgh and San Diego) will participate in the trial.

14-019.html), which do not allow foreign components, only US EMS agencies will participate in the trial.

Candidate EMS agencies will need to meet prequalification criteria to enter the enrollment phase of the trial. (Appendix 3)

12. Interventions

12.1 EMS Personnel Training Levels

The interventions in this trial may involve coordination between advanced- and basic-level EMS personnel.[57]

- Advanced-level EMS personnel (Advanced Life Support, ALS, paramedic, advanced care paramedic) perform advanced-level interventions, including intravenous drug administration, manual defibrillation, and advanced airway management interventions (ETI and SGA).
- Basic-level EMS personnel (Basic Life Support, BLS, Emergency Medical Technician, EMT) perform basic measures such as CPR and automated defibrillation. Bag-valve-mask ventilation is their primary airway management intervention, although select EMS agencies may perform SGA (LT) insertion.[58,59] In general, basic-level EMS personnel in the US do not perform ETI.

EMS organization and response to OHCA vary across ROC. In some EMS agencies, all responding personnel are trained at the advanced level. In other agencies, OHCA care is tiered, with both basic-level and advanced-level EMS personnel responding to OHCA. Select BLS EMS personnel among ROC EMS agencies perform SGA insertion (Table 2). The trial design will try to accommodate all configurations of EMS response.

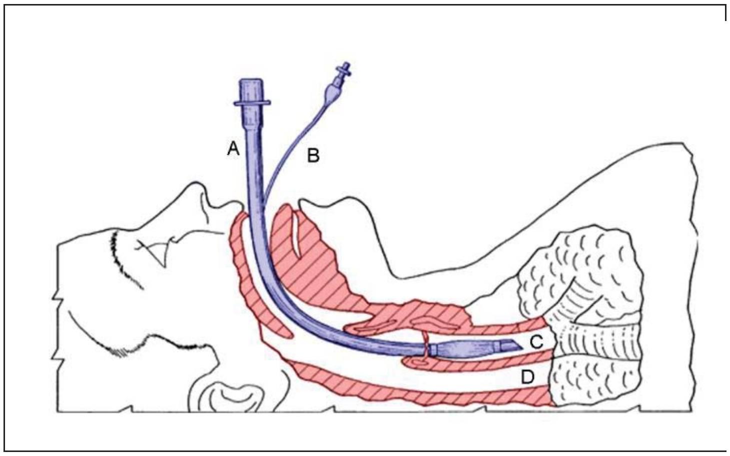

Figure 7 – Example of Endotracheal Tube
(from http://commons.wikimedia.org/wiki/Image: Endotracheal_tube_inserted.png)

12.2 Primary Endotracheal Intubation (Control Arm)

In this traditional model of OHCA airway management, advanced-level EMS personnel will use ETI as the primary (initial) airway management intervention. If the EMS agency is assigned to this arm, basic-level EMS personnel will use bag-valve-mask ventilation only even if they would normally use an LT.

12.2.1 ETI Device and Technique

ETI involves placement of a flexible plastic breathing tube between the vocal cords and into the trachea. The customary approach to ETI is through the patient's mouth (orotracheal intubation). The operator inserts a metal lighted blade (laryngoscope) into the patient's mouth to displace the

tongue and expose the vocal cords. The operator the places the ET tube directly through the vocal cords. (Figure 7)

12.2.2 ETI Techniques Permitted in the Trial

Conventional orotracheal intubation is the preferred approach for the primary/initial intubation attempt. Orotracheal ETI is the most common approach in OHCA. EMS personnel may use video laryngoscopy for the primary/initial intubation attempt.

Alternate approaches to ETI (for example, nasotracheal intubation, digital intubation) are less common in OHCA and will not be allowed for the initial intubation effort.

If the EMS agency is assigned to the primary ETI arm, and an alternate ETI technique is used for the primary/initial intubation attempt, the case will be excluded from the study.

If the EMS agency is assigned to the primary ETI arm, and if initial orotracheal ETI attempts are unsuccessful, the EMS agency may use alternate ETI techniques in a "rescue" capacity.

12.3 Primary Laryngeal Tube Insertion (Intervention Arm)

In this test model of OHCA airway management, advanced-level EMS personnel will use LT as the primary (initial) airway management intervention. Basic-level EMS personnel will use bag-valve-mask ventilation. If trained to use LT, basic-level EMS personnel may perform LT insertion.

12.3.1 LT Device and Technique

The LT is a novel airway device that is inserted blindly through the mouth. The device is designed so that the distal end sits in the proximal esophagus. A single port results in inflation of proximal (hypopharyngeal) and distal (esophageal) cuffs. Fenestrations between the cuffs allow insufflated air and oxygen to indirectly enter the tracheal. (Figure 8)

King Systems is the manufacturer and distributor of LT airways in the United States. There are several such models, including reusable (autoclavable) models as well as models with an esophageal port. We expect that EMS agencies will use only the disposable models of the King LT (LT-D and LTS-D).

Use of the LT in adults with OHCA has been described in a range of studies.[60-62]

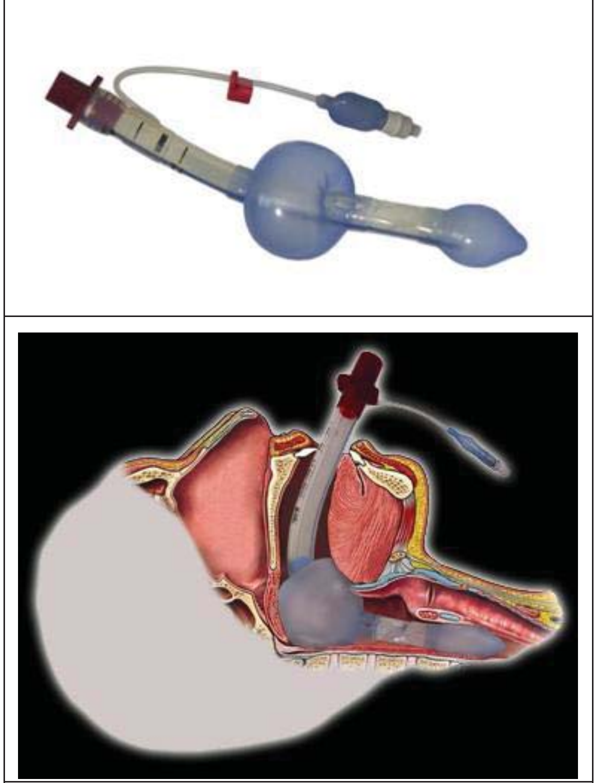

Figure 8 – Examples of Laryngeal Tube
http://www.ambuusa.com/usa/products/clinical_studies/king_lt%E2%84%A2.aspx

http://theopsdeck.com/MEDKIT%20CONTENTS/PROD%20-%20AWY.King%20LTD.PMI.htm

12.3.2 Limitation of the Trial Intervention Arm to the Laryngeal Tube

Only LT use will be permitted in the intervention arm of this trial. Combitubes, LMAs, i-Gels or other supraglottic airway devices use will not be permitted in this study.

In North America, the most common SGA in EMS use are the King Laryngeal Tube (King LT – King Systems, Noblesville, IN), Combitube (Covidien, Inc, Mansfield, MA), Laryngeal Mask Airway (LMA – LMA North America, San Diego, CA) and the i-Gel (Intersurgical, Inc., Wokingham, United Kingdom). In preparation for this trial, we have collected information on the airway management practices of 33 US EMS agencies that have actively participated in prior ROC OHCA trials and that indicated interested in participating in the trial. (Table 2) The vast majority utilize the King Laryngeal Tube. A small number of EMS agencies use the Combitube or i-Gel.

The resource limitations of this trial do not allow for subset analysis of multiple SGA devices. Therefore, only the LT will be permitted in this study.

EMS personnel may utilize other SGA devices on non-trial patients.

12.4 Research Procedures

The airway interventions specified in this trial will be implemented by EMS personnel within the context of local Basic Life Support (BLS) and Advanced Cardiac Life Support (ACLS) protocols and practices. Customary BLS interventions include assessment of pulse, initiation of chest compressions, operation of automated external defibrillator, and opening and management of the airway. Customary ACLS interventions include assessment of pulse, initiation of chest compressions, assessment of cardiac rhythm, delivery of rescue defibrillation shocks, insertion of intravenous lines, administration of drugs, and opening and management of the airway. (Figure 9) While there is general unified approach to BLS and ACLS, some EMS agencies have implemented minor modifications. For example, while some agencies perform an immediate ECG rhythm check before initiating chest compressions, others first perform 1-2 minutes of chest compressions before ECG rhythm check. Some agencies delay insertion of airway device until after initiation of intravenous line and medications.

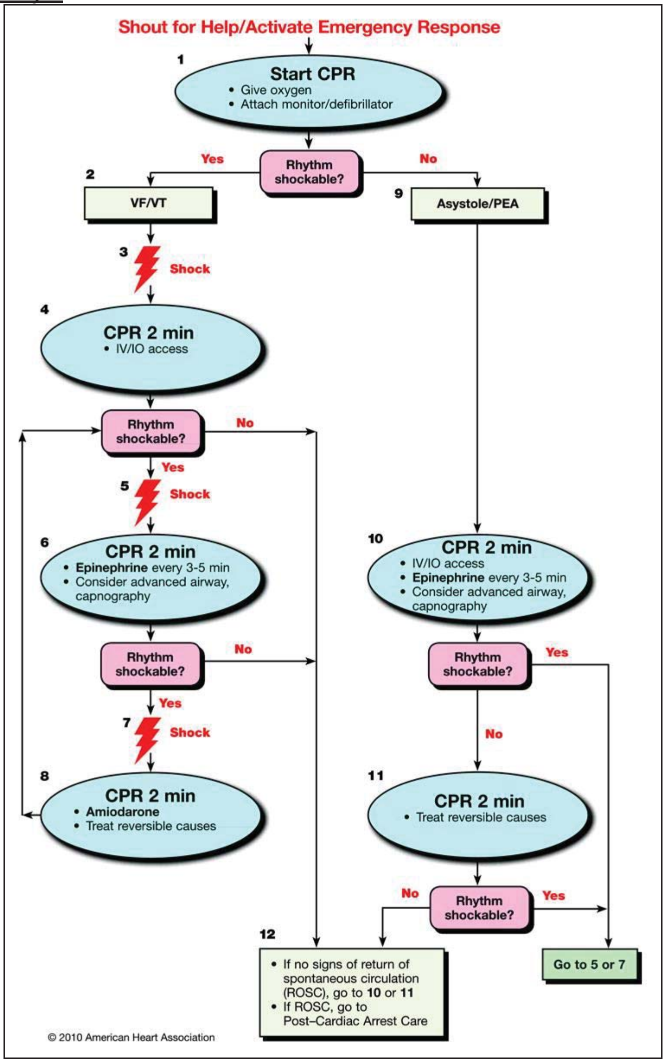

Figure 9 - American Heart Association Algorithm for Adult Cardiac Life Support
ETI or LT insertion may typically occur at boxes 6 and 10, "Consider advanced airway."[63] EMS agencies will follow local ACLS protocols.

This protocol does not dictate the timing or sequence of ETI or LT insertion. Basic- and

advanced-level EMS personnel will perform airway management (ETI, LT or BVM) during resuscitation as guided by local protocol or practice.

Upon arriving upon the scene of a potentially eligible subject, treating EMS personnel will initiate resuscitation measures per local BLS and ACLS protocol. Patient assessment, initiation and style of chest compressions, rhythm assessment, shock delivery, intravenous line insertion, and drug administration will be performed according to local protocol and practices.

Simultaneously, EMS personnel will verify eligibility for the trial (i.e., satisfaction of inclusion criteria and absence of any exclusion criteria).

At the point where they are ready to perform advanced airway insertion, EMS personnel will select the airway management technique to which they have been randomized (ETI or LT). EMS personnel will perform ETI or LT insertion using techniques consistent with local protocol.

EMS personnel will verify successful and correct placement of the ET tube or LT according to local protocols. Customary methods for confirming tube placement including physical examination (chest rise), auscultation of chest for presence of breath sounds, auscultation of epigastrium for absence of gastric sounds, use of a bulb syringe detection, use of a colorimetric end-tidal carbon dioxide detector, and use of a waveform digital end-tidal carbon dioxide detector.

EMS personnel will secure airway device in place per local protocol. Standard methods for securing the ET tube and LT airway include adhesive tape, umbilical "twill" tape and commercial tube holders.[64,65] Of note, the LT cannot be secured using an ET tube commercial tube holder; rescuers should use adhesive tube or a tube holder designed for the larger diameter of the LT.

After securing the airway device, EMS personnel will continue with standard resuscitation measures. Decisions for termination of resuscitation in the field or transporting to receiving Emergency Department are not dictated by this trial.

Upon arrival at the receiving ED, resuscitation – including management of the airway – may continue according to local practices and protocol. Resuscitation efforts will continue until terminated by ED staff. If there is restoration of spontaneous circulation, post-arrest care will proceed according to local protocols and practice.

12.5 Protocol Considerations

12.5.1 Number of Airway Insertion Attempts

Successful ETI or LT insertion may require multiple attempts. With "attempt" defined as passage of the laryngoscope blade into the mouth, Wang, et al. observed that first-attempt success for paramedic ETI of OHCA was 70%, with up to four attempts needed to reach the 90% success threshold.[6] EMS practitioners generally try to minimize the number of ETI attempts because of the propensity of oral injury and because multiple attempts may signal ETI futility and the need to proceed to alternate airway management techniques. In clinical practice, some EMS protocols limit practitioners to 3, 2 or even 1 unsuccessful ETI attempt before proceeding to alternate "rescue" airway measures.

This trial will not dictate or limit the number of ET tube or LT insertion attempts. However, we will monitor the number of insertion attempts for each device. Training for the trial will emphasize a)

definition of “ETI attempt” as insertion of laryngoscope between the teeth (not insertion of ET tube), b) definition of “LT attempt” as insertion of LT airway between the teeth, c) methods for reporting the number of attempts for each airway device, and d) strategies for minimizing ETI or LT insertion attempts.

12.5.2 Unsuccessful Airway Insertion and Rescue Interventions

An important metric in this study is the rate of unsuccessful ETI or LT insertion and the need for rescue airway interventions. While success rates for OHCA ETI and SGA insertion in OHCA are relatively high (approximately 90%), the time-critical nature of OHCA requires that rescue airway interventions be initiated promptly.[66,67] Clinical signs of airway insertion or ventilation failure include the inability to place the airway, inability to confirm proper airway device placement, detectable air leak on insufflation resulting in suboptimal chest rise.

In the event that EMS personnel are not able to insert or ventilate through the assigned airway, they may revert to any alternate airway management strategy. For example, if the patient is assigned to ETI but intubation efforts are unsuccessful, EMS personnel may revert to bag-valve-mask ventilation or LT insertion. Conversely, if the patient is assigned to LT but insertion efforts are unsuccessful, EMS personnel may revert to bag-valve-mask ventilation or ETI. In all cases, subjects will be analyzed by their assigned treatment, per intention-to-treat principles.

While select EMS agencies may carry additional SGA other than the LT, during training we will encourage the use of LT as the preferred rescue device in the event of unsuccessful ETI.

Alternate intubation techniques (nasotracheal intubation, digital intubation, etc.) may be used in a rescue capacity in the event of failed initial orotracheal intubation or LT insertion.

Some EMS agencies perform needle jet ventilation or open cricothyroidotomy. This trial does not preclude the use of these techniques in the event of unsuccessful initial airway insertion.

12.5.3 Confirmation of Airway Placement

An important adverse event of ETI is initially unrecognized misplacement of the ET tube, including unrecognized placement in the esophagus or hypopharynx. Unrecognized misplacement of the ET tube may result in suboptimal delivery of oxygen to the lungs. National consensus guidelines recommend the use of multiple techniques for conforming proper ET tube placement, including auscultation of lungs (for presence of breath sounds) and epigastrium (for absence of gastric sounds), use of an esophageal detector device (bulb detector device), use of a colorimetric end tidal carbon dioxide detector, or use of a digital or waveform end-tidal carbon dioxide detector.[68] ET tube misplacement may impact the apparent effectiveness of the ETI arm. Therefore, this trial will monitor the techniques used for verifying ET tube placement.

While this trial will not dictate ET tube placement confirmation techniques, best practices for verifying tube placement will be emphasized during training. We will similarly encourage receiving EDs to use best practices when verifying the proper placement of an EMS placed ET tube.

Airway misplacement is less of a concern for the LT because the device is intended to be placed in the esophagus. Because of the design of the LT, it is highly unusual for the device to be placed intratracheally. There are no national guidelines for verifying proper LT placement.

12.5.4 Use of Neuromuscular Blocking Agents

The use of neuromuscular blocking (NMB) agents to facilitate ETI is termed “Rapid Sequence Intubation” (RSI). Most OHCA patients are flaccid and do not require RSI. On rare occasion, an OHCA patient may develop trismus, requiring the use of RSI. RSI is technique reserved for a very small subset of specially trained paramedics. Of the ground EMS agencies in ROC that will participate in the trial, few use RSI. Also, an air medical crew capable of performing RSI may be present on scene of an OHCA.

Subjects receiving NMB (RSI) prior to (or during) ETI or LT insertion efforts will be examined in separate subgroup analyses.

Subjects receiving NMB agents after successful airway insertion will not be examined separately.

12.5.5 Absence of a Bag-Valve-Mask-Only Arm

This trial will not have a BVM-only arm. Some prior observational studies suggested higher survival with BVM-only vs ETI or SGA techniques.[41] However, many practitioners and community individuals perceive a need to provide some form of ventilatory support in OHCA. In our preparation for this trial, ROC EMS personnel indicated discomfort with a BVM-only arm. Furthermore the resource constraints of this pragmatic trial preclude the recruitment of an adequate number of subjects to have sufficient power to detect clinically important differences in a three arm trial.

12.5.6 Emergency Department Management of the EMS Airway Device

Most trial patients will be transported to a receiving ED for continued resuscitation care. There are currently no clinical practice guidelines for the ED management of an EMS-placed ET tube or SGA. In general, ED practitioners will verify placement of the EMS ET tube, although practices vary widely. If the ET tube is determined to be misplaced or insufficiently ventilating, ED physicians may opt to remove and replace the tube.

With SGA such as the LT, ED practices similarly vary, with some practitioners leaving the airway in place for the duration of resuscitation and others opting for immediate replacement with ETI. There are no national practice guidelines for the ED management of an EMS-placed SGA, although select textbook chapters offer practical guidance.[69]

In practical terms, if the EMS-placed LT is functioning well, ED practitioners may continue using the device, deferring ETI until there is restoration of pulses. In contrast, if there is evidence that the LT is malfunctioning, immediate replacement with ETI or another SGA may be indicated. Replacement of the LT with ETI may occur with a variety of approaches including exchange over a gum elastic bougie (Eschman tube), bronchoscopic replacement over an Aintree catheter, or removal and ETI using direct laryngoscopy.

The pragmatic scope of this trial will focus on out-of-hospital EMS management of the airway. This trial will not dictate practices for ED management of EMS-placed airways. However, in preparation for the trial we will provide receiving ED with guidelines on best practices for the management of EMS-placed airways. The guidelines will outline strategies for:

- ED verification of EMS-placed ET tubes, including the use of at least two confirmatory techniques as well as the use of end-tidal carbon dioxide detection.

- Decision points for immediate replacement of an EMS ET tube.

- Management of EMS-placed LT airways, including methods and decision points for continued use or replacement with an ET tube.

12.5.7 EMS Personnel Airway Management Experience

Studies highlight that EMS personnel experience may influence the course and outcomes of airway management efforts. For example, using Pennsylvania statewide EMS data we have found that paramedic ETI experience is associated with increased patient survival.[70] As described previously, opportunities for EMS airway training are limited, and paramedics in some practice settings perform few clinical ETI.[33,34]

The evaluation of paramedic airway management experience and its influence on OHCA outcomes is beyond the scope of this study. This study will not track the number of airway procedures performed by individual EMS personnel.

12.5.8 Post-Arrest Care

This study will not dictate elements of post-arrest care. Receiving hospitals will provide post-arrest care according to local protocols and practices.

Case-control studies have evaluated the effectiveness of combinations of hospital-based treatments in patients resuscitated from cardiac arrest in a variety of settings.[61,71-75] All have reported improved outcomes when compared with historical controls. Collectively, these studies demonstrate that hospital-based care of those resuscitated from OHCA impacts patient outcomes and potentially modifies the effect of prehospital interventions for cardiac arrest. Thus experts have recommended a standardized approach to try to achieve optimal outcomes after resuscitation from cardiac arrest.[76]

This pragmatic trial is not focused upon elements of post-arrest care. Given the random treatment assignment, we expect to see an equal distribution of post-arrest care practices in each study arm.

The trial will monitor the provision of key post-arrest care elements:

- **Therapeutic Hypothermia (TH) and Targeted Temperature Management (TTM)** – TH and TTM refer to the active reduction and/or control of body temperature. In the context of OHCA, TH/TTM is intended to improve outcomes through the prevention of cellular level organ damage, especially of the brain. Two randomized trials have demonstrated the safety and effectiveness of mild TH/TTM) via external cooling methods in comatose survivors of adult VF OHCA.[77,78] Another recent trial demonstrated no difference in survival or favorable neurologic status between TTM strategies using 33°C and 36°C as targeted body temperatures.[79] We will monitor the use of TH/TTM among enrolled subjects. We will define TH/TTM as a) any active attempt to lower and/or control body temperature ≤36˚C, b) continuation of temperature control efforts for at least 12 hours, and c) whether a minimum temperature <34°C is

achieved. Each of these three factors was associated with survival in a post hoc analysis of patients enrolled in the ROC PRIMED trials.

- **Percutaneous Coronary Intervention (PCI)** – Up to 71% of patients with cardiac arrest have coronary artery disease, and nearly half have an acute coronary occlusion.[80-82] There is a high incidence (97%) of coronary artery disease in patients resuscitated from OHCA who undergo immediate angiography and a 50% incidence of acute coronary occlusion.[81] We will monitor early use of PCI within 6 hours of the initial OHCA resuscitation. We will monitor the use of both coronary angiography as well as interventions (e.g., coronary artery stenting).

- **Withdrawal of Care** – Reliable prognostic factors are established after post-arrest day three, but an analysis of observational data from an in-hospital cardiac arrest registry demonstrated that the majority of declarations of do not attempt resuscitation (DNAR) status or withdrawal of life-supporting therapies occur prematurely.[83] In the ROC PRIMED Trial, the timing of withdrawal-of-care varied. (Figure 10) We will monitor the assignment and timing of physician order for 'do not resuscitate' status or withdrawal of care.

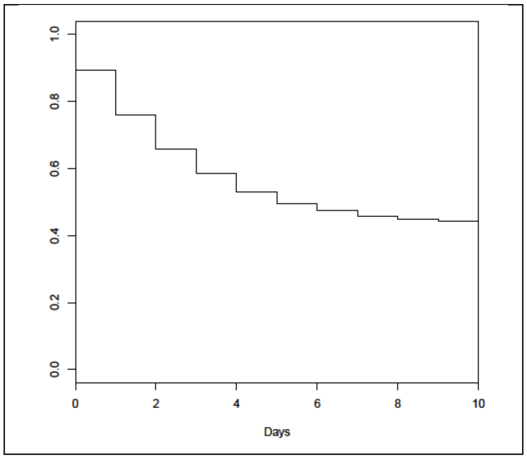

Figure 10 - Timing of Withdrawal-of-Care in the ROC PRIMED Study

13. Random Allocation and Blinding

13.1 Random Allocation Method

The airway intervention will be cluster randomized at the EMS agency level with periodic crossover. Each site will be subdivided into multiple clusters by EMS agency, station, or other unit as appropriate to the site's EMS structure. Each cluster will be scheduled to crossover to the opposite treatment – typically once or twice per year during the trial. The use of crossovers is efficient and minimizes unbalanced treatment assignment.

If more than one participating EMS agency is present on scene, the first arriving unit will determine the study treatment assignment. If a non-ROC EMS agency arrives first on scene, the subsequent first arriving ROC EMS agency will perform airway management as randomized.

The randomization of clusters will be stratified by site. Within each site, clusters will be organized into relatively homogeneous groups with respect to the number of patients expected to be treated over the course of the study in that cluster. All clusters will crossover between intervention assignments at least once (i.e., have at least two distinct treatment periods). If necessary, some clusters with high episode rates will crossover more than once.

Random assignment of treatment sequence will be performed by the coordinating center prior to the start of the study.

13.2 Rationale for Cluster Randomization

The clustered design with crossover is the most feasible, efficient and practical design in this study. In our protocol development efforts, ROC EMS personnel clearly indicated a preference for the cluster-crossover approach. ROC EMS agencies have had extensive experience with this approach in the prior PRIMED and CPR Feedback trials, and the ongoing CCC OHCA trial.[84-86]

Randomization by event would add unacceptable complexity for EMS providers who already must deal with the need for immediate therapy in OHCA. Event level randomization requires the preparation of treatment envelopes (potentially biased) or blinded airway equipment pouches, which is neither feasible nor practical given the range of different airway equipment used by EMS. Randomization by telemetry is not feasible due to the time-critical nature of OHCA and airway management and the varying systems of on-line EMS medical command throughout ROC. While the Gausche, et al. trial utilized alternate day randomization (odd day=BVM, even day=BVM±ETI), ROC EMS personnel believed that this approach would be difficult and prone to protocol violation.[45] The ongoing UK airway trial led by Benger, et al. is randomizing subjects by paramedic.[48] This strategy may result in selection bias and can be problematic if multiple study paramedics (each assigned to different arms) are delivering care.

13.3 Blinding of Treatment Assignment

This trial will use cluster randomization techniques. Furthermore, the type of airway device used by EMS personnel cannot be concealed. Therefore, blinding of the random assignment is not possible.

14. Outcomes

14.1 Primary Outcome

14.1.1 72-Hour Survival

The primary outcome of the trial is **72-hour hospital survival**, defined as patient status (alive/dead) at 72 hours after the initial onset of cardiac arrest.

While studies of OHCA are traditionally designed to evaluate neurologically-intact survival to hospital discharge, the use of 72-hour survival as the primary outcome is consistent with the pragmatic focus of this trial:[88]

- **Pragmatic Focus and Interpretation** – A pragmatic trial focuses on real-world clinical endpoints, with less attention paid to secondary, explanatory or mechanistic endpoints. Some EMS and hospital practitioners believe that out-of-hospital interventions have more plausible connections with proximal outcomes (such as 72-hour survival) rather than later outcomes (such as neurologically intact survival to hospital discharge).

- **Sample Size** – The use of 72-hour survival requires far fewer subjects than more distal outcomes such as neurologically-intact survival to hospital discharge (<3,000 vs. >18,000

- subjects). A larger targeted enrollment is not feasible given the resource limitations for this trial.

- **Associations at 72 hours are expected to be similar to those observed at later time points** – In a prior study of 1,496 adult OHCA in Pittsburgh, we observed that the vast majority of OHCA deaths (90.6%) occurred within three days.[87] (Figure 11) In our post hoc analysis of airway management practices in the ROC PRIMED trials, associations between 24-hour survival and airway type (ETI or SGA) persisted for neurologically intact survival to hospital discharge. (Figure 4)

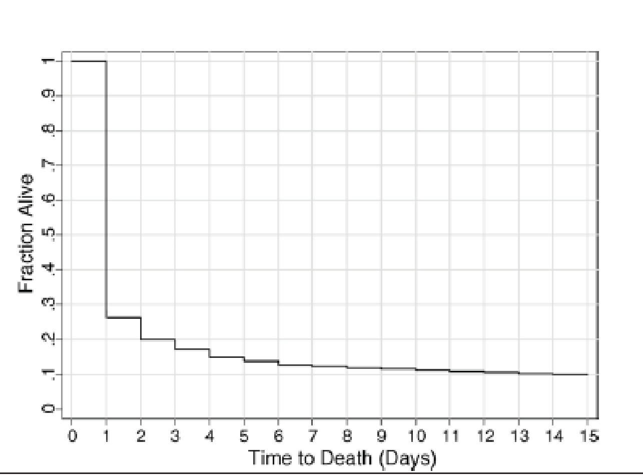

Figure 11 - Survival curve of 1,496 adult OHCA (Pittsburgh, PA).
Most (90.6%) deaths occurred within 3 days (72 hours) of the cardiac arrest event.[87]

- **Accommodates therapeutic hypothermia and care withdrawal window** – A commonly assessed outcome in OHCA is 24-hour survival. In this trial we have extended this window to 72 hours because many OHCA patients receive therapeutic hypothermia, a process that requires 48-72 hours.[89] Also, national guidelines for cardiac arrest care suggest that withdraw of care should not be considered until at least 72 hours after the initial cardiac arrest event.[90]

14.2 Secondary Outcomes

Secondary trial outcomes will include:

14.2.1 Return of Spontaneous Circulation

ROSC will be defined as the presence of palpable pulses upon ED arrival.

14.2.2 Airway Management Course

To characterize the course of airway management, this study will collect a limited number of airway management variables.

- **Sequence and success of ETI/LT insertion attempts.** EMS personnel will report the sequence and success of airway devices insertion efforts. Potential airway intervention combinations are summarized in Table 2.

First (Randomly Assigned) Airway Intervention	Second "Rescue" Airway Intervention (if first unsuccessful)	Third "Rescue" Airway Intervention (if second unsuccessful)
ETI<br>LT	ETI<br>LT<br>BVM<br>Other Airway (including alternate ETI techniques)	BVM<br>Other Airway (including alternate ETI techniques)

Table 2 – Potential EMS Airway Intervention Combinations

- **Number of airway insertion attempts.** For each subject, EMS personnel will separately report the

number of ETI attempts (successful or unsuccessful) and number of LT attempts (successful or unsuccessful).

- **Successful airway insertion time.** EMS personnel will report the time of successful ETI or LT insertion.

- **Airway course in receiving Emergency Department.** The purpose of these data is to ascertain how the receiving ED utilizes airway devices placed by EMS personnel. Research coordinators will review ED records to determine the course of airway management during initial ED resuscitation. For cases arriving at the ED with ongoing CPR, this time period encompasses the time elapsed until the achievement of ED ROSC. For cases arriving at the ED with ROSC, this time period encompasses the first 20 minutes in the ED.

  These data do not pertain to airway interventions performed after the achievement of ROSC (for those arriving with ongoing CPR) or after the first 20 minutes in the ED (for those arriving at the ED with ROSC). Potential EMS/ED airway interventions are summarized in Table 3.

EMS Airway Intervention on ED Arrival	ED Airway Intervention
ETI<br>LT<br>BVM<br>Other Airway	Continue use of EMS airway<br>Replace with ED ETI<br>Replace with other ED airway

Table 3 – Potential Emergency Department Airway Interventions

14.2.3 Survival to Hospital Discharge

While this study is not designed to evaluate associations with survival to hospital discharge, we will nonetheless monitor and assess this outcome.

14.2.4 Neurologically-Intact Survival to Hospital Discharge

While this study is not intended to have adequate power to detect associations with neurologically-intact survival to hospital discharge, we will nonetheless monitor and assess this outcome. MRS has concurrent validity with other measures of neurological recovery after stroke and brain injury.[91,92] MRS has been previously used in a cohort of neurosurgical patients with in-hospital cardiac arrest,[93] a cohort of survivors of OHCA,[94] and a cohort of survivors of arrest in either setting.[95]

Neurologic status at discharge will be assessed using the modified Rankin Score (MRS), which can be determined through medical record review.[96,97] The MRS uses a seven-point ordinal scale; 0 (no symptoms at all) to 6 (death). Patients who die before discharge will be assigned an MRS of 6. For analytic purposes, MRS at discharge is often dichotomized to MRS ≤3 (“good”) vs. 4-6 (“poor”).[98,99]

14.3 Adverse Events

The trial will identify a range of out-of-hospital and in-hospital adverse events information to help assess the safety of the interventions.

14.3.1 Unexpected Adverse Events (UAE)

These will be defined as any serious unexpected adverse effect on health or safety or any unexpected life-threatening problem caused by, or associated with the interventions if that effect or problem was not previously identified in nature, severity, or degree of incidence in the investigation plan or application (including a supplementary plan or application), or any other unexpected serious problem that relates to the rights, safety or welfare of subjects. The death or neurological impairment of an individual patient is not considered an unexpected adverse event in this study.

14.3.2 Expected Adverse Events

The following are events commonly observed in patients who experience cardiac arrest or resuscitative efforts after its onset, or who receive airway management efforts, and may or may not be attributable to specific resuscitation therapies.[4,11,100-108] These events will be monitored and reported but not considered as adverse events of the study intervention.

- **Unsuccessful EMS airway insertion.** This event includes unsuccessful EMS ETI or LT insertion efforts. This event does not include ED airway insertion efforts. This event will be determined from EMS records.

- **Multiple EMS airway insertion attempts.** This event includes 3 or more EMS attempts to perform ETI, or 3 or more EMS attempts to insert the LT. This event does not include ED airway management efforts. This event will be determined from EMS records.

- **Unrecognized EMS airway misplacement or dislodgement.** This event includes unrecognized EMS endotracheal tube placement in the esophagus or hypopharynx, or displacement of a correctly placed ET tube. Airway misplacement and dislodgements are difficult to differentiate; this trial will not differentiate these events. Airway misplacement/dislodgement events will be determined from EMS and ED records.

  This event does not include right mainstem intubations. This event does not include instances where EMS personnel immediately recognize and correct ET tube misplacement. This event does not include ED airway misplacements.

  Misplacement and dislodgement events are unique to ETI and do not apply to the LT.

- **Inadequate EMS ventilation.** This event includes instances where EMS personnel a) perceive that a successfully inserted airway device is resulting in inadequate ventilation and b) therefore opt to change to another airway management device or technique. Inadequate ventilation connotes a) inability to insufflate any oxygen (airway obstruction) or b) inadequate chest rise on insufflation. Potential reasons for inadequate ventilation may include device obstruction, misplacement, malfunction or cuff leak, among others.

  Inadequate ventilation will be based upon EMS personnel assessment, and will be determined from EMS patient care records.

- **Airway swelling or edema.** A described complication of the LT is the development of major tongue, pharyngeal or hypopharyngeal swelling, which may interfere with ventilation.[109] While usually associated with instances of prolonged LT ventilation (several hours), some clinicians have reported the occurrence of this event during resuscitative efforts. This event

will be based upon EMS and ED personnel assessment and will be determined from EMS and ED patient care records. The timeframe for surveillance for this event will be limited to the earlier of a) replacement of the LT with an ET tube, or b) the first 24 hours of hospitalization. While generally associated with LT insertion, we will monitor the occurrence of airway swelling or edema regardless of inserted airway device.

- **Oropharyngeal or hypopharyngeal injury.** Advanced airway insertion may result in injury to the oropharynx and hypopharynx, including soft tissue lacerations, injury to teeth, and perforation of pharyngeal, hypopharyngeal or other anatomic structures, among others. This event will be determined by review of the discharge summary and ED physician records and will be limited to injuries identified during the first 24 hours of hospitalization.

- **Pneumothorax.** Airway insertion and ventilatory efforts may cause pulmonary barotrauma, resulting in pneumothorax. This event will be determined from radiology or ED physician interpretation of the first chest x-ray or computed tomography (CT) performed in the ED.

- **Pneumonia and aspiration pneumonitis.** Pneumonia and aspiration pneumonitis are known sequelae of cardiac arrest and airway management. This event will be defined as the presence of an infiltrate or consolidation as reported by radiology interpretation of chest x-rays or computed tomography (CT) performed during the first 72 hours of hospitalization.

14.4 Other Data

Additional data will be collected from the out-of-hospital, Emergency Department and hospital care phases.

14.4.1 CPR Process Data

All participating ROC EMS units have automated external defibrillators (AED) and/or manual monitor/defibrillators capable of monitoring individual components of CPR process; for example, the delivery of individual chest compressions, chest compression rate, interruptions, and fraction, and (in select cases) chest compression depth. Currently, all CPR process data files are uploaded to the UW CTC. While this archiving process will continue during the study (in order to allow for rhythm audits), CPR process data analysis will not be part of this trial. However, additional grants may provide funding for the review of the electronic ECG, entry of data into the database, analysis of CPR process data and selected audits.

14.4.2 Other Explanatory Mechanisms

Physiologic mechanisms hypothesized to influence OHCA outcomes after paramedic airway management include CPR interruptions, hyperventilation, and carotid artery impingement.[7,20,21,51] This trial focuses on pragmatic outcomes and will not examine these mechanisms.

15. Analysis Plan

15.1 CONSORT Diagram

Figure 12 provides the anticipated CONSORT Diagram for the trial. The trial will exclude OHCA that do not meet inclusion criteria, as well as those who do not require ventilatory support. The remaining subjects will be enrolled and randomized in the trial.

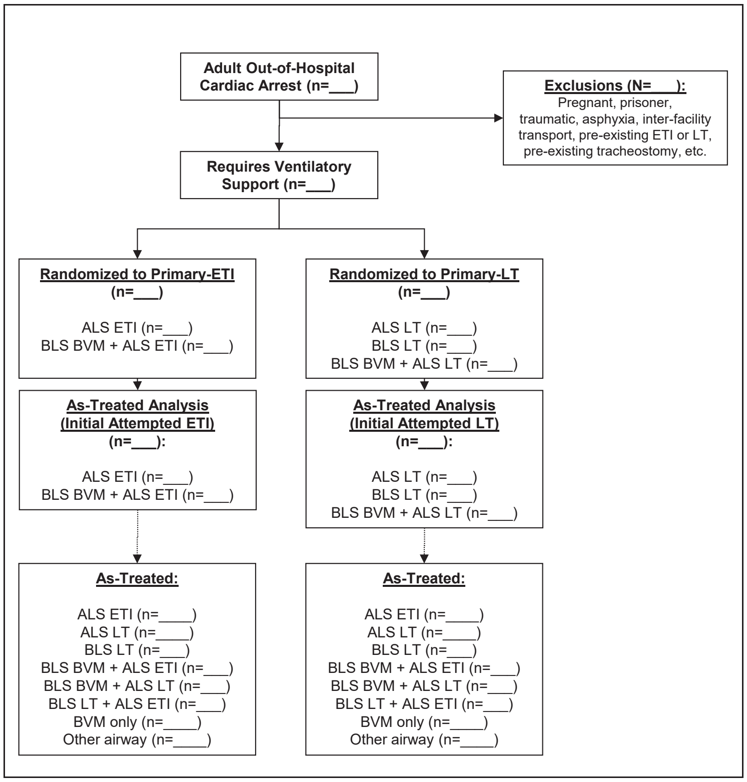

Figure 12 - Anticipated CONSORT Diagram

15.2 Intention-to-Treat vs As-Treated

The intention-to-treat comparison will include the two treatment arms: primary-ETI and primary-LT. Where allowed by local protocol, the primary-LT arm will allow for BLS LT insertion.

The as-treated comparison will encompass the initial advanced airway technique received by the subject, whether the airway insertion is successful or unsuccessful. Practically, this will account for situations where randomization is violated; for example, use of the ETI when the patient is randomized to primary-LT, or BLS rescuer use of LT when the patient is assigned to primary-ETI (and hence BLS BVM). In cases where rescuers use only BVM (without resorting to ETI or LT insertion), this will not be considered a protocol violation, and the subject will be retained in the assigned randomization. In cases where rescuers use a different technique as the initial airway intervention (other than ETI or LT), the subject will be excluded from the as-treated analysis.

While this trial focuses on two airway management strategies (primary-ETI and primary-LT), in execution the course of airway management may encompass other airway management endpoints, including BVM ventilation, use of ETI other than orotracheal technique, use of a SG other than LT, or use of an another airway management technique (for example, cricothyroidotomy). Also, airway insertion efforts may prove unsuccessful, necessitating the use of alternate airway techniques. Therefore, a second as-treated comparison will consider the final result of EMS airway management efforts: a) ETI, b) LT, c) BVM, and d) other airway technique.

15.3 Primary Analysis – 72-Hour Survival

The primary hypothesis is that there is no difference in OHCA 72-hour survival between primary LT- and primary-ETI airway management strategies. The primary outcome of interest is 72-hour survival. We will calculate the difference in outcomes divided by the estimated "robust" standard error based on the Huber-White sandwich estimator in order to account for within cluster correlation and variability, which might depart from the classical assumptions.[12] To quantify the treatment effect, we will calculate the 95% CI for the difference in event rates. Since

randomization includes all eligible OHCA patients, we will not stratify the primary analysis by initial cardiac rhythm. We will perform the analysis on intention-to-treat (primary) as well as as-treated (secondary) bases.

15.4 Secondary Analyses

In a similar manner, we will also study associations between the trial interventions and the secondary outcomes detailed in Section 14.2, including return of spontaneous circulation, airway management course, adverse events, hospital survival, and hospital survival with good neurologic function.

15.5 Subgroup Analyses

OHCA outcomes are known to vary for different patient subsets. For example, OHCA survival is approximately 28.4% for patients with an initial “shockable” cardiac rhythms (ventricular fibrillation or tachycardia) and 6.2% for non-shockable rhythms (pulseless electrical activity and asystole). We will explore multiplicative associations between airway management strategy (ETI vs. LT) and the pertinent subgroups, including:

- Initial cardiac rhythm (shockable vs. non-shockable);
- Bystander witnessed arrest (yes vs. no);
- EMS response time (911 call to arrival on-scene: <10 vs. ≥10 mins);
- BLS unit capability of performing LT insertion (yes vs. no);
- Time of airway placement after rescuer arrival on-scene (early [<10 minutes] vs. later [≥10 minutes]);
- Use of NMB (RSI) before or during airway insertion efforts (yes vs no);
- Age (<65 vs. ≥65 years).
- Placement of advanced airway after ROSC.
- Initial airway management with non-rebreather mask only (passive ventilation).

Initial airway management with video laryngoscopy. If an [airway x subgroup] interaction is statistically significant, we will stratify the analysis by the subgroup.

16. <u>Sample Size</u>

16.1 Sample Size Estimate

The sample size estimate is based upon the primary outcome of 72-hour survival. Table 4 provides total sample size requirements to detect effect differences

	**Statistical Power (1-β)**		
**Effect Size (Absolute Difference)**	**80%**	**85%**	**90%**
**4.0%**	2,856	3,266	3,892
**4.5%**	2,284	**2,612**	3,112
**5.0%**	1,872	2,142	2,550

Table 4 - Sample Size Estimates
Effect sizes based upon 13.7% baseline 72-hour survival.

in 72-hour survival after OHCA, with an overall significance level (adjusted for interim analyses) of 0.05 and accommodating up to a 5% loss of precision due to cluster randomization (with crossover).

The estimated effect sizes are based upon observations from the ROC PRIMED Trial. In ROC PRIMED, baseline 72-hour survival was 13.7%. There was a 5.0% absolute difference in 72-hour survival between ETI (13.7%) and SGA (8.7%). To account for potential confounders, for this trial we will use a more conservative figure of 4.5% to represent a minimal clinically significant difference. We designed the trial to have 85% power to detect this difference. Therefore, the required minimum sample size is 2,612 subjects (1,306 per group). To allow for subject withdrawal and loss to follow-up, we have budgeted to enroll up to a total of 3,000 subjects.

The participating US ROC sites (Alabama, Dallas, Milwaukee, Pittsburgh, Portland and San Diego) historically treated over 3,000 OHCA requiring advanced airway management in 2013. Therefore, we expect to have adequate capacity to enroll the requisite number of subjects over the anticipated three-year study period.

16.2 Interim Monitoring Plan and Stopping Boundaries – Criteria for Terminating the Trial

With input from the Data Safety and Monitoring Board (DSMB), prior to initiation of the trial, we will develop a monitoring plan to guide early termination of the trial. Factors influencing stopping decision may include (a) formal stopping rules based upon the primary analysis, (b) information on safety outcomes by treatment group, (c) consistency between results for primary and secondary outcomes, and (d) consistency of treatment effects across subgroups.

			Lower Stopping Boundary (SGA Better)			
**Analysis**	**Cum. Sample Size**	**Prop. Max Stat Info**	**Absolute Difference**	**Adjusted Difference**	**95% Confidence Interval**	**P-value**
1	654	0.25	-0.108	-0.098	(-0.154, -0.041)	0.001
2	1,306	0.50	-0.102	-0.092	(-0.135, -0.053)	<0.001
3	1,958	0.75	-0.092	-0.084	(-0.120, -0.052)	<0.001
4	2,612	1.00	-0.029	-0.029	(-0.059, 0.000)	0.050

			Upper Stopping Boundary (ETI Better)			
**Analysis**	**Cum. Sample Size**	**Prop. Max Stat Info**	**Absolute Difference**	**Adjusted Difference**	**95% Confidence Interval**	**P-value**
1	654	0.25	0.125	0.116	(0.058, 0.172)	<0.001
2	1,306	0.50	0.118	0.109	(0.069, 0.151)	<0.001
3	1,958	0.75	0.106	0.099	(0.066, 0.135)	<0.001
4	2,612	1.00	0.029	0.029	(0.000, 0.059)	0.050

Table 5 - Interim Stopping Boundaries for 72-Hour Survival

The formal stopping boundaries are asymmetric, two-sided designs which are included in the unified family of group sequential stopping rules.[110,111] (Table 5) The boundaries are asymmetric, because the utility/ease of use/training for a SGA is judged to be preferable to ETI. The tests for

superiority of either intervention will be based upon boundaries with parameters P=(0,0), R=(0.2,0.2), A=(0.355,0.29), and epsilon=c(1,1), with a two-sided overall alpha level of 0.05.[79]

We envision conducting 4 total analyses (3 interim + 1 final). The study does not include any futility boundaries because obtaining estimates in either direction will be informative; even an estimate that both treatments yield similar 72-hour survival is informative.

At the conclusion of the clinical trial, reported point estimates, 95% confidence intervals, and p-values for the primary outcome will be adjusted for the true sampling distribution accounting for the stopping rule. Point estimates from interim analyses will be based on the bias adjusted point estimate.[112] Confidence intervals and p-values will be calculated from the ordering of the outcome space based upon the maximum likelihood estimate.[113] The stopping rules described above can be implemented using S+SeqTrial (S+SEQTRIAL User's Manual, Insightful, Inc., Seattle WA, 2000).

17. Eligibility for Participating and Remaining in the Enrollment Phase of the Trial

Prior to entering the enrollment phase of the trial, each EMS agency must demonstrate readiness to participate in the study. EMS agencies must also meet select compliance benchmarks to remain the enrollment phase of the trial. Specific criteria for participating and remaining in the enrollment phase are listed in Appendix 3.

The trial will not have a formal run-in phase.

The study leadership will appoint a Study Monitoring Committee (SMC - Section 22) to oversee and review EMS agency study compliance. The SMC will meet on a bi-monthly basis to review enrollment and protocol adherence. The SMC will have the authority to recommend removal of an EMS agency from trial participation for protocol or data collection noncompliance.

18. Timeline

18.1 Milestones for the Planning Phase

The Year 1 planning phase of this trial is scheduled for September 15, 2014 - July 30, 2015. The parameters of the grant award for this trial specify that the certain milestones must be met during the Year 1 planning phase prior to proceeding to the Year 2-5 trial execution/subject enrollment phase.

Tasks that will be completed during the Year 1 planning phase include:

- Develop and finalize trial protocol;
- Register trial at www.clinicaltrials.gov;
- Obtain Food and Drug Administration (FDA) review for Investigational Device Exemption (IDE);
- Finalize selection and definition of data elements;
- Program and test data base;

- Obtain initial Institutional Review Board (IRB) approval;
- Initiate Exception from Informed Consent (EFIC) community consultation and public disclosure (CC/PD);
- Develop and test EMS training materials;
- Establish site subawards.

A program evaluation will be performed by an independent agency appointed by NHLBI.

18.2 Milestones for Trial Execution and Subject Recruitment

Trial execution and subject recruitment will occur over the 4-year period August 1, 2015 – July 30, 2019.

We expect 6 ROC regional coordinating centers encompassing over 33 EMS agencies to participate in the trial. We expect participating EMS agencies to complete a brief period (2-4 months) of data collection to demonstrate readiness for the enrollment phase of the trial. We expect recruitment of subjects to occur through July 30, 2018 (3 years). Trial close-out and analysis will occur during August 1, 2018 – July 30, 2019.

19. Data Sources and Management

19.1 Data Sources

Data will be collected prospectively as patient care progresses. This will include a review of all EMS and hospital records.

19.1.1 Out-of-Hospital EMS Data

Out-of-hospital data will be determined from EMS clinical care documentation, including EMS dispatch records and patient care records.

CPR process files will be collected and archived but will not be analyzed as part of the primary trial effort (although additional grant funding may support this effort in the future).

19.1.2 Hospital Data

Hospital records that will be reviewed for this trial may include:

- Emergency Department and inpatient physician clinical documentation
- Emergency Department and inpatient nurse clinical documentation
- Radiology reports
- Operative reports
- Procedural notes
- Discharge summary
- Death note and certificate

19.1.3 Post-Discharge Vital Status

This trial will not require the determination of post-discharge vital status.

19.2 Data Entry

The DCC will provide web-based HTML forms to collect necessary information from the participating sites. Web entry forms will have dynamic features such as immediate checks on data and relationships within a form and between forms. Details and clarification about data items will be provided using pop-up windows and links to appropriate sections of the on-line version of the Manual of Operations. Data encryption and authentication methods will be used. The DCC will build additional features into the web entry forms including: forms transmission history, access to past forms, tracking of data corrections, and the capability to save and re-load incomplete forms.

19.3 Database Management

The DCC will use a two-tiered database structure. A front-end database will serve the web entry needs, using a database management system well-suited to handling updates from multiple interactive users. The data from this database will be transferred periodically (e.g., weekly) to a data repository that can be used by statistical software packages. These data sets will be the basis for data queries, analyses and monitoring reports. Various versions of this database will be kept as needed, e.g., for quarterly performance reports. Backup of data and programs will be performed at frequent intervals. Access to data will be limited to those who need access to perform their tasks. The database management system is able to manage large quantities of data, to merge data from multiple databases as required, to handle complex and possibly changing relationships, and to produce analysis datasets that can be imported into a variety of statistical analysis packages.

20. EMS Research Protocol Training

20.1 Overview of EMS Protocol Training Elements

Training of EMS personnel for this trial will encompass didactic and practical modalities. The salient components of EMS personnel training are described below. While we will develop a master set of training materials, individual regional coordinating centers will be permitted to customize the format to suit local needs.

Prior to entering the trial, participating EMS agencies will summarize their experience with and standard training practices (format, frequency) for both airway devices.

All participating EMS agencies will receive initial training in the trial protocol. Key elements of training will include:

- **Scientific Basis for the Trial –** This section include level-appropriate presentation of the scientific principles of airway management, a summary of prior studies of OHCA airway management, and the rationale for the current randomized trial. All EMS personnel that may potentially carry out study procedures will be required to undergo training.

- **Review of Study Protocol –** This section will include the following: overall study design, inclusion and exclusion criteria, the process of exception to informed consent under

emergency circumstances, the study protocol and procedures, data reporting, and contingency measures.

- **Protocol Practicum –** EMS personnel will have the opportunity to simulate execution of the trial protocol in "hands-on" fashion. This practical experience will encompass the use of mannequins that can accommodate ETI and LT insertion. EMS personnel will be given the opportunity to practice to proficiency each component of the protocol. EMS personnel will have the opportunity to integrate the trial procedures in the context of other ACLS interventions. Various permutations of the study protocol will be presented, including each of the study arms and scenarios with unsuccessful initial airway attempts.

- **Data Reporting –** Select data elements specific to airway management are required for this trial; for example, the sequence of airway interventions, the number of attempts, the timing of successful airway insertion, and adverse events. The training will review definitions and reporting practices for each of these data elements.

- **Cognitive Post-Test –** A cognitive post-test will cover key trial procedures and may be completed online or as a written or verbal component of the training sessions. A record of training completion will be maintained by each site or EMS agency.

20.2 Retraining and Feedback

All EMS agencies will receiving periodic training in trial procedures. Retraining will focus on proper execution of research procedures and reporting of data. Where possible, retraining will occur before each crossover period and as required by the ROC Study Monitoring Committee.

In keeping with the pragmatic design of the trial, the trial will not prescribe the frequency, methods or modalities for training in airway management techniques. EMS agencies will provide airway management skills training using their existing practices.

EMS personnel will receive study performance feedback throughout the trial.

21. Guidance for Receiving Emergency Departments

The practices and procedures employed by receiving Emergency Departments will not be dictated by this trial. However, to each potential receiving ED we will provide information on best ED practices for managing EMS-placed airways. This information will include:

- **Overview of the Trial –** We will provide an overview of the rationale for the trial and the trial interventions. We will provide an overview of the LT airway and its function.

- **Best Practices for Confirming EMS ET Tube Placement –** Best practices for confirming EMS ET tube placement will be reviewed. Emphasis will be placed on a) the need for using multiple modalities for confirming ET tube placement, b) the use of waveform capnography as a preferred modality for confirming tube placement, c) the use of direct laryngoscopic revisualization when placement is uncertain. Emphasis will be placed on the preference of maintaining EMS ET tube use over emergent ED re-intubation.

- **Best Practices and Strategies for Managing EMS-Inserted LT Airways –** Best practices for confirming EMS LT airway placement will be reviewed. The guidelines will encourage

using of the EMS LT for ongoing resuscitation, with conversion to ETI deferred until ROSC. Strategies for LT conversion to ETI will be reviewed, including direct laryngoscopy and intubation, exchange of LT to ET tube over a bougie, exchange of LT to ET tube over an Aintree catheter, and cricothyroidotomy or tracheotomy.

22. Study Monitoring

22.1 Study Monitoring Committee

The study leadership will appoint a Study Monitoring Committee (SMC) to oversee EMS participation and performance in the trial. The SMC will include, at minimum, the PI, the lead co-investigators, select members of the DCC, select members of the trial workgroup, select PIs from participating sites, and select representatives of NIH. The SMC will work with the study leadership to develop criteria for EMS agencies to enter and remain in the enrollment phase of the trial. Based upon reports provided by the DCC, the SMC will monitor EMS agency enrollment performance on a bi-monthly basis.

The SMC will have the final authority to authorize EMS agency participation in and/or suspension from participation in the trial.

22.2 Data Safety and Monitoring Board

ROC has an existing independent Data Safety and Monitoring Board (DSMB) that will monitor adverse events throughout the trial and review outcomes data for possible harm. The DSMB was appointed by NHLBI. The committee will review and approve the protocol before the study commences. In addition, the committee will approve an interim monitoring plan before study initiation and review the results of the interim analyses. Although the DSMB will make the final decision about the interim monitoring plan, we anticipate that the DSMB will evaluate treatment compliance and the rate of adverse events between the treatment and control arms at intervals to be determined by the DSMB, expected to be approximately semi-annually. The DSMB will also monitor primary, secondary and mechanistic study outcomes between the treatment and control groups including main effects and a priori subgroups as specified elsewhere in the protocol. The DSMB will advise the investigators if a change in the protocol is warranted based on this interim monitoring.

The DCC will forward DSMB reports to study investigators, the Institutional Review Board, and the sponsor in accordance with the 1996 guidance from OHRP regulations 46.101 (i), as is our current practice.

Clinical staff will report all potential adverse events to the coordinating center as soon as possible. These will be collected in both a structured (standard form) and open (describing any difficulties encountered) form. All potential adverse events will be reviewed as to treatment arm and further classified by: a) Severity (life-threatening, serious, non-serious); and b) Expected vs. Unexpected. Expected adverse events will be recorded as noted in the hospital discharge summary by each enrolling site, reported to overseeing agencies as required by federal regulations and local requirements, and reviewed periodically by our independent data safety monitoring board.

For suspected unexpected serious adverse reactions (SUSAR), the coordinating center will promptly notify the DSMB as well as appropriate regulatory agencies, site and sponsor. The coordinating

center will tabulate and report compliance, data quality, and non-serious adverse events on a regular basis.

The SMC will carry out recommendations provided by the DSMB.

23. Regulatory Considerations

23.1 Human Subjects

Because of the unconscious nature of cardiac arrest victims and the time-critical nature of OHCA care, the trial will be carried out under "Exception from informed consent required for emergency research" (EFIC) rules outlined in by the guidance document "Waiver of Informed Consent Requirements in Certain Emergency Research" (Federal Register, Vol. 61, pp. 51531-51533, November 1, 1996 - http://www.gpo.gov/fdsys/pkg/FR-1996-10-02/html/96-24968.htm), OHRP "Basic HHS Policy for Protection of Human Research Subjects" (45 CFR 46.101(i) - http://www.hhs.gov/ohrp/humansubjects/guidance/45cfr46.html#46.101), and FDA regulations 21 CFR 50.24.[84,85,114]

The use of EFIC is justified because the victims of OHCA are in a life-threatening situation, available OHCA treatments are unproven or unsatisfactory, and obtaining informed consent prior to enrollment is not feasible because of their unconscious and critically-ill state, and trial interventions must be administered before consent from the subjects' legally authorized representatives is feasible. Cardiac arrest is a short-lived illness, for which immediate interventions cannot be delayed without irreparable harm to the patient.

In conformance with EFIC rules, measures to protect the rights and welfare of subjects will include, at least; a) community consultation, b) public disclosure, c) independent data monitoring, and d) notification of a legally authorized representative or family members of a subject's enrollment in the trial, with opportunity to discontinue trial participation.

Additional details are provided in Appendix 4. Suggested notification documents are provided in Appendix 5.

23.2 Food and Drug Administration

Based upon review of a formal application submitted by the study team, the FDA confirmed that the trial is *exempt* from FDA Investigational Device Exemption (IDE) regulations. (Communication from FDA to Henry Wang, MD, January 8, 2015.)

23.2.1 Technical and Regulatory Information for the ET Tube

The ET tube is listed in the FDA Medical Devices Database under the device name "Tube, Tracheal (w/wo connector)" and is classified as Device Class 2.

23.2.2 Technical and Regulatory Information for the LT

Models of the King LT airway are listed in the FDA Medical Devices Database. (Table 6) The predicate device is “Airway, Oropharyngeal, Anesthesiology.” There is no 510(K) number for the King LTS-D model, as it is considered a minimal modification of the LT-D and LTS models. We expect that only disposable versions of the King LT will be used in this study.

Manufacturer	FDA 510(K) #	Models
KING SYSTEMS, CORP NOBLESVILLE, IN	K021634	KING LT, MODELS # KLT 100, KLT 101, KLT 102, KLT 103, KLT 104, KLT 105
	K033186	KING LT-D DISPOSABLE OROPHARYNGEAL AIRWAY, MODELS KLT 203, KLT 204 AND KLT 205
	K033189	KING LTS REUSABLE OROPHARYNGEAL AIRWAY WITH DRAIN/ /SUCTION CHANNEL, MODELS KLT 303, KLT 304 AND KLT 305

Table 6 – King LT Models and Corresponding FDA 510(K) Numbers

24. References

1. Go AS, Mozaffarian D, Roger VL, et al. Heart Disease and Stroke Statistics--2014 Update: A Report From the American Heart Association. Circulation 2013.

2. Kette F, Reffo I, Giordani G, et al. The use of laryngeal tube by nurses in out-of-hospital emergencies: preliminary experience. Resuscitation 2005;66:21-5.

3. Khan SA, Siddiqui MM, Khan RM. Laryngoceles and the laryngeal tube. Anaesthesia 2003;58:391.

4. Bein B, Carstensen S, Gleim M, et al. A comparison of the proseal laryngeal mask airway, the laryngeal tube S and the oesophageal-tracheal combitube during routine surgical procedures. European journal of anaesthesiology 2005;22:341-6.

5. Dunford JV, Davis DP, Ochs M, Doney M, Hoyt DB. Incidence of transient hypoxia and pulse rate reactivity during paramedic rapid sequence intubation. Annals of emergency medicine 2003;42:721-8.

6. Lee JS, Nam SB, Chang CH, Han DW, Lee YW, Shin CS. Relationship between arterial and end-tidal carbon dioxide pressures during anesthesia using a laryngeal tube. Acta anaesthesiologica Scandinavica 2005;49:759-62.

7. Kurola J, Pere P, Niemi-Murola L, et al. Comparison of airway management with the intubating laryngeal mask, laryngeal tube and CobraPLA by paramedical students in anaesthetized patients. Acta anaesthesiologica Scandinavica 2006;50:40-4.

8. Richebe P, Verdonck O, Biais M, Breton Y, Cros AM, Maurette P. [Clinical evaluation of the single use Laryngeal Tube in adults: the LTD]. Annales francaises d'anesthesie et de reanimation 2005;24:1250-4.

9. Asai T, Moriyama S, Nishita Y, Kawachi S. Use of the laryngeal tube during cardiopulmonary resuscitation by paramedical staff. Anaesthesia 2003;58:393-4.

10. Wang HE, Szydlo D, Stouffer JA, et al. Endotracheal intubation versus supraglottic airway insertion in out-of-hospital cardiac arrest. Resuscitation 2012;83:1061-6.

11. Turan A, Kaya G, Koyuncu O, Karamanlioglu B, Pamukcu Z. Comparison of the laryngeal mask (LMA) and laryngeal tube (LT) with the new perilaryngeal airway (CobraPLA) in short surgical procedures. European journal of anaesthesiology 2006;23:234-8.

12. White H. Maximum likelihood estimation of misspecified models. Econometrica 1982;50:1-25.

13. Wang HE, Kupas DF, Greenwood MJ, et al. An algorithmic approach to prehospital airway management. Prehospital emergency care : official journal of the National Association of EMS Physicians and the National Association of State EMS Directors 2005;9:145-55.

14. DeLeo BC. Endotracheal intubation by rescue squad personnel. Heart Lung 1977;6:851-4.

15. Guss DA, Posluszny M. Paramedic orotracheal intubation: a feasibility study. Am J Emerg Med 1984;2:399-401.

16. Jacobs LM, Berrizbeitia LD, Bennett B, Madigan C. Endotracheal intubation in the prehospital phase of emergency medical care. Jama 1983;250:2175-7.

17. Stewart RD, Paris PM, Winter PM, Pelton GH, Cannon GM. Field endotracheal intubation by paramedical personnel. Success rates and complications. Chest 1984;85:341-5.

18. Katz SH, Falk JL. Misplaced endotracheal tubes by paramedics in an urban emergency medical services system. Annals of emergency medicine 2001;37:32-7.

19. Wang HE, Mann NC, Mears G, Jacobson K, Yealy DM. Out-of-hospital airway management in the United States. Resuscitation 2011;82:378-85.

20. Aufderheide TP, Lurie KG. Death by hyperventilation: a common and life-threatening problem during cardiopulmonary resuscitation. Critical care medicine 2004;32:S345-51.

21. Aufderheide TP, Sigurdsson G, Pirrallo RG, et al. Hyperventilation-induced hypotension during cardiopulmonary resuscitation. Circulation 2004;109:1960-5.

22. Paradis NA, Martin GB, Rivers EP, et al. Coronary perfusion pressure and the return of spontaneous circulation in human cardiopulmonary resuscitation. Jama 1990;263:1106-13.

23. Cheskes S, Schmicker RH, Christenson J, et al. Perishock pause: an independent predictor of survival from out-of-hospital shockable cardiac arrest. Circulation 2011;124:58-66.

24. Christenson J, Andrusiek D, Everson-Stewart S, et al. Chest compression fraction determines survival in patients with out-of-hospital ventricular fibrillation. Circulation 2009;120:1241-7.

25. Roth H, Genzwuerker HV, Rothhaas A, Finteis T, Schmeck J. The ProSeal laryngeal mask airway and the laryngeal tube Suction for ventilation in gynaecological patients undergoing laparoscopic surgery. European journal of anaesthesiology 2005;22:117-22.

26. National Highway Traffic Safety Administration. Emergency Medical Technician Paramedic:National Standard Curriculum (EMT-P). 1998. (Accessed Oct 8, 2004, at http://www.nhtsa.dot.gov/people/injury/ems/EMT-P/.)

27. de Oliveira Filho GR. The construction of learning curves for basic skills in anesthetic procedures: an application for the cumulative sum method. Anesth Analg 2002;95:411-6, table of contents.

28. Accreditation Council for Graduate Medical Education: Emergency Medicine - Guidelines for Procedures and Resuscitations. 2004. (Accessed November 12, 2004, at http://www.acgme.org/acWebsite/RRC_110/110_guidelines.asp#res.)

29. Council on Accreditation of Nurse Anesthesia Educational Programs: Standards for Accreditation of Nurse Anesthesia Educational Programs. Park Ridge, Illinois; 2004.

30. Konrad C, Schupfer G, Wietlisbach M, Gerber H. Learning manual skills in anesthesiology: Is there a recommended number of cases for anesthetic procedures? Anesth Analg 1998;86:635-9.

31. Kopacz DJ, Neal JM, Pollock JE. The regional anesthesia "learning curve". What is the minimum number of epidural and spinal blocks to reach consistency? Reg Anesth 1996;21:182-90.

32. Charuluxananan S, Kyokong O, Somboonviboon W, Pothimamaka S. Learning manual skills in spinal anesthesia and orotracheal intubation: is there any recommended number of cases for anesthesia residency training program? J Med Assoc Thai 2001;84 Suppl 1:S251-5.

33. Wang HE, Kupas DF, Hostler D, Cooney R, Yealy DM, Lave JR. Procedural experience with out-of-hospital endotracheal intubation. Crit Care Med 2005;33:1718-21.

34. Burton JH, Baumann MR, Maoz T, Bradshaw JR, Lebrun JE. Endotracheal intubation in a rural EMS state: procedure utilization and impact of skills maintenance guidelines. Prehosp Emerg Care 2003;7:352-6.

35. Gabrielli A, Layon AJ, Wenzel V, Dorges V, Idris AH. Alternative ventilation strategies in cardiopulmonary resuscitation. Curr Opin Crit Care 2002;8:199-211.

36. Hagberg C, Bogomolny Y, Gilmore C, Gibson V, Kaitner M, Khurana S. An evaluation of the insertion and function of a new supraglottic airway device, the King LT, during spontaneous ventilation. Anesth Analg 2006;102:621-5.

37. Guyette FX, Greenwood MJ, Neubecker D, Roth R, Wang HE. Alternate airways in the prehospital setting (resource document to NAEMSP position statement). Prehosp Emerg Care 2007;11:56-61.

38. Fales W, Farrell R. Impact of New Resuscitation Guidelines on Out-of-Hospital Cardiac Arrest Survival (abstract). Academic Emergency Medicine 2007;14:S157-S8.

39. McMullan JT, McNally B, Gerecht R, Bonomo J, Robb R, Wang HE. Airway Management and Outcomes After Out of Hospital Cardiac Arrest in the CARES Network (abstract). Circulation 2013;128:A9545.

40. Hanif MA, Kaji AH, Niemann JT. Advanced airway management does not improve outcome of out-of-hospital cardiac arrest. Academic emergency medicine : official journal of the Society for Academic Emergency Medicine 2010;17:926-31.

41. Hasegawa K, Hiraide A, Chang Y, Brown DF. Association of prehospital advanced airway management with neurologic outcome and survival in patients with out-of-hospital cardiac arrest. JAMA : the journal of the American Medical Association 2013;309:257-66.

42. Studnek JR, Thestrup L, Vandeventer S, et al. The association between prehospital endotracheal intubation attempts and survival to hospital discharge among out-of-hospital cardiac arrest patients. Academic emergency medicine : official journal of the Society for Academic Emergency Medicine 2010;17:918-25.

43. Tanabe S, Ogawa T, Akahane M, et al. Comparison of neurological outcome between tracheal intubation and supraglottic airway device insertion of out-of-hospital cardiac arrest patients: a nationwide, population-based, observational study. The Journal of emergency medicine 2013;44:389-97.

44. Kajino K, Iwami T, Kitamura T, et al. Comparison of supraglottic airway versus endotracheal intubation for the pre-hospital treatment of out-of-hospital cardiac arrest. Critical care 2011;15:R236.

45. Gausche M, Lewis RJ, Stratton SJ, et al. Effect of out-of-hospital pediatric endotracheal intubation on survival and neurological outcome: a controlled clinical trial. JAMA : the journal of the American Medical Association 2000;283:783-90.

46. Bernard SA, Nguyen V, Cameron P, et al. Prehospital rapid sequence intubation improves functional outcome for patients with severe traumatic brain injury: a randomized controlled trial. Annals of surgery 2010;252:959-65.

47. Frascone RJ, Russi C, Lick C, et al. Comparison of prehospital insertion success rates and time to insertion between standard endotracheal intubation and a supraglottic airway. Resuscitation 2011;82:1529-36.

48. Benger JR, Voss S, Coates D, et al. Randomised comparison of the effectiveness of the laryngeal mask airway supreme, i-gel and current practice in the initial airway management of prehospital cardiac arrest (REVIVE-Airways): a feasibility study research protocol. BMJ open 2013;3.

49. Birkholz T, Irouschek A, Kessler P, Blunk JA, Labahn D, Schmidt J. Feasibility of the laryngeal tube airway for artificial ventilation in pigs and comparison with the laryngeal mask airway. Lab animal 2008;37:371-9.

50. Yamamoto Y, Inoue S, Abe R, Kawaguchi M, Furuya H. Airway management with the laryngeal tube in rabbits. Lab animal 2007;36:33-5.

51. Segal N, Yannopoulos D, Mahoney BD, et al. Impairment of carotid artery blood flow by supraglottic airway use in a swine model of cardiac arrest. Resuscitation 2012;83:1025-30.

52. Carlson JN, Reynolds JC. Does advanced airway management improve outcomes in adult out-of-hospital cardiac arrest? Annals of emergency medicine 2014;64:163-4.

53. Fouche PF, Simpson PM, Bendall J, Thomas RE, Cone DC, Doi SA. Airways in out-of-hospital cardiac arrest: systematic review and meta-analysis. Prehospital emergency care : official journal of the National Association of EMS Physicians and the National Association of State EMS Directors 2014;18:244-56.

54. McMullan J, Gerecht R, Bonomo J, et al. Airway management and out-of-hospital cardiac arrest outcome in the CARES registry. Resuscitation 2014;85:617-22.

55. Shin SD, Ahn KO, Song KJ, Park CB, Lee EJ. Out-of-hospital airway management and cardiac arrest outcomes: a propensity score matched analysis. Resuscitation 2012;83:313-9.

56. Ocker H, Wenzel V, Schmucker P, Steinfath M, Dorges V. A comparison of the laryngeal tube with the laryngeal mask airway during routine surgical procedures. Anesthesia and analgesia 2002;95:1094-7, table of contents.

57. Davis DP, Garberson LA, Andrusiek DL, et al. A descriptive analysis of Emergency Medical Service Systems participating in the Resuscitation Outcomes Consortium (ROC) network. Prehospital emergency care : official journal of the National Association of EMS Physicians and the National Association of State EMS Directors 2007;11:369-82.

58. Ochs M, Vilke GM, Chan TC, Moats T, Buchanan J. Successful prehospital airway management by EMT-Ds using the combitube. Prehosp Emerg Care 2000;4:333-7.

59. Cady CE, Pirrallo RG. The effect of Combitube use on paramedic experience in endotracheal intubation. Am J Emerg Med 2005;23:868-71.

60. Bernhard M, Beres W, Timmermann A, et al. Prehospital airway management using the laryngeal tube. An emergency department point of view. Der Anaesthesist 2014;63:589-96.

61. Sunde K, Pytte M, Jacobsen D, et al. Implementation of a standardised treatment protocol for post resuscitation care after out-of-hospital cardiac arrest. Resuscitation 2007;73:29-39.

62. Gruber E, Oberhammer R, Balkenhol K, et al. Basic life support trained nurses ventilate more efficiently with laryngeal mask supreme than with facemask or laryngeal tube suction-disposable--a prospective, randomized clinical trial. Resuscitation 2014;85:499-502.

63. Neumar RW, Otto CW, Link MS, et al. Part 8: Adult Advanced Cardiovascular Life Support: 2010 American Heart Association Guidelines for Cardiopulmonary Resuscitation and Emergency Cardiovascular Care. Circulation 2010;122:S729-S67.

64. Cook TM, McCormick B, Asai T. Randomized comparison of laryngeal tube with classic laryngeal mask airway for anaesthesia with controlled ventilation. British journal of anaesthesia 2003;91:373-8.

65. Khan SA, Siddiqi MM, Khan RM. Diffusion of nitrous oxide into the cuff of the laryngeal

tube. Anaesthesia 2003;58:291.

66. Hubble MW, Brown L, Wilfong DA, Hertelendy A, Benner RW, Richards ME. A meta-analysis of prehospital airway control techniques part I: orotracheal and nasotracheal intubation success rates. Prehospital emergency care : official journal of the National Association of EMS Physicians and the National Association of State EMS Directors 2010;14:377-401.

67. Hubble MW, Wilfong DA, Brown LH, Hertelendy A, Benner RW. A meta-analysis of prehospital airway control techniques part II: alternative airway devices and cricothyrotomy success rates. Prehospital emergency care : official journal of the National Association of EMS Physicians and the National Association of State EMS Directors 2010;14:515-30.

68. Cook TM, McKinstry C, Hardy R, Twigg S. Randomized crossover comparison of the ProSeal laryngeal mask airway with the Laryngeal Tube during anaesthesia with controlled ventilation. British journal of anaesthesia 2003;91:678-83.

69. Wang HE, Thomsen DP. Airway Management. In: Cone DC, Brice JH, Delbridge TR, Myers JB, eds. Emergency medical services : clinical practice and systems oversight. Second edition. ed: Kendall/Hunt; 2009:p.383-403.

70. Chiu CL, Murugasu J, Chan L. The use of modified VBM laryngeal tube compared to Laryngeal Mask Airway during spontaneous ventilation. Anaesthesia and intensive care 2003;31:187-92.

71. Wolcke BB, Mauer DK, Schoefmann MF, et al. Comparison of standard cardiopulmonary resuscitation versus the combination of active compression-decompression cardiopulmonary resuscitation and an inspiratory impedance threshold device for out-of-hospital cardiac arrest. Circulation 2003;108:2201-5.

72. Oddo M, Schaller MD, Feihl F, Ribordy V, Liaudet L. From evidence to clinical practice: effective implementation of therapeutic hypothermia to improve patient outcome after cardiac arrest. Critical care medicine 2006;34:1865-73.

73. Knafelj R, Radsel P, Ploj T, Noc M. Primary percutaneous coronary intervention and mild induced hypothermia in comatose survivors of ventricular fibrillation with ST-elevation acute myocardial infarction. Resuscitation 2007;74:227-34.

74. Wolfrum S, Pierau C, Radke PW, Schunkert H, Kurowski V. Mild therapeutic hypothermia in patients after out-of-hospital cardiac arrest due to acute ST-segment elevation myocardial infarction undergoing immediate percutaneous coronary intervention. Critical care medicine 2008;36:1780-6.

75. Rittenberger JC, Guyette FX, Tisherman SA, DeVita MA, Alvarez RJ, Callaway CW. Outcomes of a hospital-wide plan to improve care of comatose survivors of cardiac arrest. Resuscitation 2008;79:198-204.

76. Gaieski DF, Band RA, Abella BS, et al. Early goal-directed hemodynamic optimization combined with therapeutic hypothermia in comatose survivors of out-of-hospital

cardiac arrest. Resuscitation 2009;80:418-24.

77. Bernard SA, Gray TW, Buist MD, et al. Treatment of comatose survivors of out-of-hospital cardiac arrest with induced hypothermia. The New England journal of medicine 2002;346:557-63.

78. Group HaCAS. Mild therapeutic hypothermia to improve the neurologic outcome after cardiac arrest. The New England journal of medicine 2002;346:549-56.

79. Asai T, Kawashima A, Hidaka I, Kawachi S. The laryngeal tube compared with the laryngeal mask: insertion, gas leak pressure and gastric insufflation. British journal of anaesthesia 2002;89:729-32.

80. Laver S, Farrow C, Turner D, Nolan J. Mode of death after admission to an intensive care unit following cardiac arrest. Intensive care medicine 2004;30:2126-8.

81. Spaulding CM, Joly LM, Rosenberg A, et al. Immediate coronary angiography in survivors of out-of-hospital cardiac arrest. The New England journal of medicine 1997;336:1629-33.

82. Pell JP, Sirel JM, Marsden AK, Ford I, Walker NL, Cobbe SM. Presentation, management, and outcome of out of hospital cardiopulmonary arrest: comparison by underlying aetiology. Heart 2003;89:839-42.

83. Vollmer T, Genzwuerker HV, Ellinger K. Fibreoptic control of the laryngeal tube position. European journal of anaesthesiology 2002;19:306-7.

84. Aufderheide TP, Nichol G, Rea TD, et al. A trial of an impedance threshold device in out-of-hospital cardiac arrest. The New England journal of medicine 2011;365:798-806.

85. Stiell IG, Nichol G, Leroux BG, et al. Early versus later rhythm analysis in patients with out-of-hospital cardiac arrest. The New England journal of medicine 2011;365:787-97.

86. Hostler D, Everson-Stewart S, Rea TD, et al. Effect of real-time feedback during cardiopulmonary resuscitation outside hospital: prospective, cluster-randomised trial. Bmj 2011;342:d512.

87. Wang HE, Min A, Hostler D, Chang CC, Callaway CW. Differential effects of out-of-hospital interventions on short- and long-term survival after cardiopulmonary arrest. Resuscitation 2005;67:69-74.

88. Chamberlain D, Cummins RO. Recommended guidelines for uniform reporting of data from out-of-hospital cardiac arrest: the 'Utstein style'. European Resuscitation Council, American Heart Association, Heart and Stroke Foundation of Canada and Australian Resuscitation Council. European journal of anaesthesiology 1992;9:245-56.

89. Holzer M. Targeted temperature management for comatose survivors of cardiac arrest. The New England journal of medicine 2010;363:1256-64.

90. Wijdicks EF, Hijdra A, Young GB, Bassetti CL, Wiebe S, Quality Standards Subcommittee of the American Academy of N. Practice parameter: prediction of outcome in comatose survivors after cardiopulmonary resuscitation (an evidence-based review): report of the Quality Standards Subcommittee of the American Academy of Neurology. Neurology 2006;67:203-10.

91. Hop JW, Rinkel GJ, Algra A, van Gijn J. Changes in functional outcome and quality of life in patients and caregivers after aneurysmal subarachnoid hemorrhage. Journal of neurosurgery 2001;95:957-63.

92. Weimar C, Kurth T, Kraywinkel K, et al. Assessment of functioning and disability after ischemic stroke. Stroke; a journal of cerebral circulation 2002;33:2053-9.

93. Rabinstein AA, McClelland RL, Wijdicks EF, Manno EM, Atkinson JL. Cardiopulmonary resuscitation in critically ill neurologic-neurosurgical patients. Mayo Clinic proceedings 2004;79:1391-5.

94. van Alem AP, de Vos R, Schmand B, Koster RW. Cognitive impairment in survivors of out-of-hospital cardiac arrest. American heart journal 2004;148:416-21.

95. Raina KD, Callaway C, Rittenberger JC, Holm MB. Neurological and functional status following cardiac arrest: method and tool utility. Resuscitation 2008;79:249-56.

96. Newcommon NJ, Green TL, Haley E, Cooke T, Hill MD. Improving the assessment of outcomes in stroke: use of a structured interview to assign grades on the modified Rankin Scale. Stroke; a journal of cerebral circulation 2003;34:377-8; author reply -8.

97. Merino JG, Lattimore SU, Warach S. Telephone assessment of stroke outcome is reliable. Stroke; a journal of cerebral circulation 2005;36:232-3.

98. Aufderheide TP, Kudenchuk PJ, Hedges JR, et al. Resuscitation Outcomes Consortium (ROC) PRIMED cardiac arrest trial methods part 1: rationale and methodology for the impedance threshold device (ITD) protocol. Resuscitation 2008;78:179-85.

99. Stiell IG, Callaway C, Davis D, et al. Resuscitation Outcomes Consortium (ROC) PRIMED cardiac arrest trial methods part 2: rationale and methodology for "Analyze Later vs. Analyze Early" protocol. Resuscitation 2008;78:186-95.

100. Baubin M, Rabl W, Pfeiffer KP, Benzer A, Gilly H. Chest injuries after active compression-decompression cardiopulmonary resuscitation (ACD-CPR) in cadavers. Resuscitation 1999;43:9-15.

101. Baubin M, Sumann G, Rabl W, Eibl G, Wenzel V, Mair P. Increased frequency of thorax injuries with ACD-CPR. Resuscitation 1999;41:33-8.

102. Huang YT, Ravi Kumar AS. Classical skeletal injuries shown on 18F-FDG PET/CT following successful cardiopulmonary resuscitation. Clinical nuclear medicine 2013;38:395-6.

DO NOT REPRODUCE WITHOUT PERMISSION

103. Cho SH, Kim EY, Choi SJ, et al. Multidetector CT and radiographic findings of lung injuries secondary to cardiopulmonary resuscitation. Injury 2013;44:1204-7.

104. Smekal D, Hansen T, Sandler H, Rubertsson S. Comparison of computed tomography and autopsy in detection of injuries after unsuccessful cardiopulmonary resuscitation. Resuscitation 2013;84:357-60.

105. Kim EY, Yang HJ, Sung YM, et al. Multidetector CT findings of skeletal chest injuries secondary to cardiopulmonary resuscitation. Resuscitation 2011;82:1285-8.

106. Hoke RS, Chamberlain D. Skeletal chest injuries secondary to cardiopulmonary resuscitation. Resuscitation 2004;63:327-38.

107. Bush CM, Jones JS, Cohle SD, Johnson H. Pediatric injuries from cardiopulmonary resuscitation. Annals of emergency medicine 1996;28:40-4.

108. Powner DJ, Holcombe PA, Mello LA. Cardiopulmonary resuscitation-related injuries. Critical care medicine 1984;12:54-5.

109. Gaither JB, Matheson J, Eberhardt A, Colwell CB. Tongue engorgement associated with prolonged use of the King-LT laryngeal tube device. Annals of emergency medicine 2010;55:367-9.

110. Kittelson JM, Emerson SS. A unifying family of group sequential test designs. Biometrics 1999;55:874-82.

111. Pampallona S, Tsiatis AA. Group sequential designs for one-sided and two-sided hypothesis testing with provision for early stopping in favor of the null hypothesis. J Stat Planning and Inference 1994;42:19-35.

112. Whitehead J. On the bias of maximum likelihood estimation following a sequential test. Biometrika 1986;73:573-81.

113. Emerson S, Fleming T. Parameter estimation following group sequetial hypothesis testing. Biometrika 1990;77:875-92.

114. Tisherman SA, Powell JL, Schmidt TA, et al. Regulatory challenges for the resuscitation outcomes consortium. Circulation 2008;118:1585-92.

25. Appendix 1 – Summary of Protocol Amendments

25.1 Version 4.2, December 10, 2014

- Results of initial protocol development.
- Protocol version sent to FDA.

25.2 Version 5.0, February 19, 2015

- Revisions made in response to DSMB comments
- Removal of duplicate inclusion/exclusion criteria
- Modification of EFIC references from FDA to OHRP rules.
- Approved by DSMB on March 4, 2015

25.3 Version 5.1, April 1, 2015

- Minor revision of exclusion criteria to exclude patients receiving initial care by non-PART participating EMS agency capable of performing ETI, LT or other advanced airway insertion. Minor revision to inclusion criteria simplifying inclusion criteria for use of bag-valve-mask.

25.4 Version 6.0, July 13, 2016

- Modifications to permit NRB in lieu of BVM as part of inclusion criteria, if allowed by local protocol.
- Clarification of chest CT as an alternative to chest x-ray.
- Clarification of hospital survival and hospital survival with good neurologic function as defined secondary outcomes.
- Updates to list of planned subgroup analyses.
- Updates to list of key study personnel.

26. Appendix 2 – List of Study Personnel

26.1 Principal Investigator, Lead Facilitator

Henry E. Wang, MD, MS
Professor and Vice Chair for Research
Department of Emergency Medicine
University of Alabama School of Medicine
(205)-996-6526/(205)-410-1267
hwang@uabmc.edu

26.2 Co-Investigators

Graham Nichol, MD
Professor, Division of General Internal Medicine
Medical Director, ROC Clinical Trial Center
University of Washington
206-685-1302
nichol@uw.edu

Susanne May, PhD
Professor
Principal Investigator
ROC Clinical Trial Center
University of Washington
206-685-1302
sjmay@uw.edu

26.3 Liaisons with the Resuscitation Outcomes Consortium

Myron Weisfeldt, MD (ROC Study Chair)
Professor
Department of Medicine
Johns Hopkins University
mlw5@jhmi.edu

Joseph P. Ornato, MD (ROC Cardiac Chair)
Professor and Chair
Department of Emergency Medicine
Virginia Commonwealth University
ornato@aol.com

Mo Daya, MD (ROC Principal Investigators)
Professor
Department of Emergency Medicine
Oregon Health Sciences University
mdaya@ohsu.edu

Ken Sternig, NREMT-P (ROC EMS Operations)
Milwaukee County EMS
Kenneth.Sternig@milwcnty.com

Dana Zive (ROC Research Coordinators)
Research Sr. Instructor
Center for Policy and Research in Emergency Medicine
Department of Emergency Medicine
Oregon Health Sciences University
zived@ohsu.edu

Pam Owens (ROC EMS Operations)
Department of Emergency Medicine
University of Texas Southwestern
pamela.owens@utsouthwestern.edu

Donna Kelly (ROC Research Coordinators)
University of California San Diego
dlkelly@ucsd.edu

26.4 Project Direction and Coordination

Shannon Stephens, NREMT-P
Project Director
Department of Emergency Medicine
University of Alabama at Birmingham
205-934-5890
swstephens@uabmc.edu

Michelle Doyle, RN, MHA
Deputy Director
ROC Clinical Trial Center
University of Washington
206-616-0417
mdoyle4@uw.edu

Heather Herren, RN, MPH
Cardiac Project Manager
ROC Clinical Trial Center
University of Washington
206-685-1302
hherren@uw.edu

26.5 Biostatisticians

Robert Schmicker, MS
ROC Clinical Trial Center

University of Washington
206-616-0434
rschmick@uw.edu

David Prince, MS
ROC Clinical Trial Center
University of Washington
206-685-1302
dprince3@uw.edu

26.6 Liaisons with the National Heart, Lung and Blood Institute

George Sopko, MD, MPH
Project Officer
Division of Cardiovascular Sciences
Heart Failure and Arrhythmias Branch
National Heart, Lung and Blood Institute
National Institutes of Health
301-435-0504
sopkog@nhlbi.nih.gov

Debra Egan, M.Sc., M.P.H.
Clinical Trial Specialist/Contractor
Division of Cardiovascular Sciences
Heart Failure and Arrhythmias Branch
National Heart, Lung and Blood Institute
National Institutes of Health
301-435-6333
debrae@nhlbi.nih.gov

27. Appendix 3 – Criteria to Enter and Remain in the Enrollment Phase of the Trial

27.1 Criteria to Enter Enrollment Phase of Trial

The following are preliminary criteria only. Final criteria will be developed and approved by the SMC and DSMB.

- Completion of written agreement to enter the trial.
- Completion of Federal Wide Assurance with sponsoring institution and Institutional Review Board.
- Verification of measures for collecting airway data elements required for the trial.
- Verification of LT as the primary SGA for use in the trial. Verification of LT availability on all EMS units. Description of supply and re-supply plan for LT used in the trial.
- Verification of protocol training for all EMS personnel.
- Provision of trial information and ED airway best practices guidelines to all receiving Emergency Departments.
- Case notification and data entry within 72 hours of the event.
- Correct reporting of all required EMS data elements, with <10% missing entries.
- Correct reporting of all required hospital data elements, with <10% missing entries.

27.2 Criteria to Remain in Enrollment Phase of Trial

This section will be finalized by the DSMB and SMC.

To remain in the trial “evaluable” phase, EMS agencies must complete all of the following requirements on either [1) 70% of eligible cases (determined by cumulative or 60-day running window), or 2) 3 consecutive cases.]

- Case notification and data entry within 72 hours.
- Correct execution of protocol, with attention to conformance with assigned airway intervention.
- Correct reporting of all required EMS data elements, with <10% missing entries.
- Correct reporting of all required hospital data elements, with <10% missing entries.

EMS agencies that are suspended from the trial may re-enter the trial by achieving the “Criteria to Enter the Active Evaluable Phase” requirements.

28. Appendix 4 – Additional Information Justifying the Use of Exception from Informed Consent (EFIC) for Emergency Research

We have outlined below how the study design applies to federal criteria for EFIC (http://www.hhs.gov/ohrp/policy/hsdc97-01.html).

(1) The human subjects are in a life-threatening situation, available treatments are unproven or unsatisfactory, and the collection of valid scientific evidence, which may include evidence obtained through randomized placebo-controlled investigations, is necessary to determine the safety and effectiveness of particular interventions.

The proposed trial is a multicenter pragmatic randomized clinical trial comparing ETI- versus SGA-airway management in patients with non-traumatic out-of-hospital cardiac arrest. These patients are in an immediate life-threatening situation with a mortality approaching 90%. The standard of care for prehospital management of these patients includes the timely provision of CPR and advanced life support including airway management.

Endotracheal intubation (ETI) is the insertion of a plastic breathing tube through the mouth and into the trachea. As the initial provider of critical care in the field, US paramedics have performed ETI on OHCA victims for over 30 years.[14-17] However, numerous studies highlight the pitfalls associated with paramedic ETI.

For example, unrecognized misplacement of the endotracheal tube (in the esophagus rather than trachea) has been reported in up to one-fourth of paramedic ETI.[18] While the endotracheal tube should ideally be placed on the first attempt, in a large series of 1,271 OHCA, we found that one-fourth of ETI efforts required two or more attempts.[6] While select studies report paramedic ETI success rates >90%, using US national data we observed ETI success rates of only 70%.[19] Paramedic ETI often leads to subsequent iatrogenic hyperventilation, which decreases coronary blood flow during CPR.[20-22]

Interruptions in CPR chest compressions – including even brief 20-second interruptions – may disrupt coronary blood flow, impairing OHCA survival.[23,24] Using state-of-the-art cardiac monitor technology, we were able to identify chest compression interruptions associated with intubation efforts by City of Pittsburgh paramedics. In this series of 100 OHCA, ETI efforts resulted in a median of 2 CPR interruptions (IQR 1-3; range 1-6) totaling a median of 109.5 seconds (IQR: 54-198; range 13-446).[7] One-fourth of the CPR interruptions exceeded three minutes.

While ETI is complex, opportunities for baseline training and continuing experience are inadequate.[9,25] Compared with the 35-200 ETI required for emergency medicine residents, anesthesiology residents and nurse anesthetist trainees, US paramedic student must perform only five (5) ETI prior to graduation.[26-32] While the customary setting for learning ETI is in the operating room training under the tutelage of anesthesiologists, students at nationally accredited paramedic training programs receive only 2-4 days of this training experience.[8] Some training programs are not able to provide any operating room training. Mannequins do not adequate recreate the “feel” of live human airways.

Regular clinical experience is important for maintaining ETI skill. Using 2003 statewide data from Pennsylvania, we found that paramedics performed a median of 1 ETI (interquartile range 0-3)

annually; 39% performed no ETI, and 67% performed fewer than two ETI.[33] In Maine, Burton, et al. found that only 40% of paramedics attempted ETI annually, and only 1-2% of all paramedics performed five or more ETI annually.[34]

Supraglottic airways (SGA) offer simpler alternatives to ETI. SGA in current North American EMS use include the Laryngeal Mask Airway (LMA), Combitube and King Laryngeal Tube.[35,36] SGA are inserted "blindly" through the mouth and sit outside of the vocal cords.[37] (Figure 3) Compared with ETI, SGAs offer simple and expeditious operation, lower skill acquisition and maintenance thresholds, and similar ventilatory characteristics. While typically reserved for contingency use in the event of unsuccessful ETI efforts, many EMS agencies perform primary SGA insertion.[38] While there are no national data descriptions, we are aware of this practice in Kalamazoo, Michigan, Chesterfield County, Virginia, Collier County, Florida, and Dallas, Texas.

We propose a large randomized trial focused on evaluation of these two interventions in the out-of-hospital cardiac arrest population, with sufficient statistical power to detect changes in outcome.

(2) Obtaining informed consent is not feasible because:

- **i. The subjects will not be able to give their informed consent as a result of their medical condition;**
- **ii. The intervention under investigation must be administered before consent from the subjects' legally authorized representatives is feasible; and**
- **iii. There is no reasonable way to identify prospectively the individuals likely to become eligible for participation in the clinical investigation.**

The study interventions need to be administered as an early intervention after the onset of cardiac arrest. In this uncontrolled setting the patient is unconscious and unable to provide consent for study enrollment. Legal next-of-kin are often not immediately available at the scene, nor is it practical for the prehospital provider to explain the study and receive consent while caring for the cardiac arrest patient. Since we are studying out-of-hospital cardiac arrest, which is frequently the first manifestation of cardiovascular disease, there is no way to prospectively identify individuals who are likely to become eligible for this trial.

(3) Participation in the research holds out the prospect of direct benefit to the subjects because:

- **i. Subjects are facing a life-threatening situation that necessitates intervention;**
- **ii. Appropriate animal and other preclinical studies have been conducted, and the information derived from those studies and related evidence support the potential for the intervention to provide a direct benefit to the individual subjects; and**
- **iii. Risks associated with the investigation are reasonable in relation to what is known about the medical condition of the potential class of subjects, the**

risks and benefits of standard therapy, if any, and what is known about the risks and benefits of the proposed intervention or activity.

As defined, these patients with cardiac arrest are facing a life-threatening situation that requires immediate intervention.

Previous animal and human studies have been conducted, and suggest the potential for a direct benefit to individual subjects in cardiac arrest via improved hemodynamics and short-term survival advantage.

The risks associated with the investigation are reasonable in relation to what is known about the medical condition of the candidate subjects, the risks and benefits of standard therapy. While airway management is an important priority in OHCA resuscitation, the best airway strategy is unknown. Compared with SGA, ETI has enormous system-level costs, including increased training and skills maintenance burden, and the heightened risk of serious adverse events (for example, tube misplacement or dislodgement). If SGAs achieves superior outcomes than ETI, this would indicate that EMS systems should switch to SGAs for OHCA resuscitation. If ETI proves superior to SGA, this would prompt efforts to optimize current ETI practices. If there is no difference in OHCA outcomes, this would affirm that EMS practitioners can select airway devices that best suit their preferences or practice setting.

(4) The clinical investigation could not practicably be carried out without the waiver.

This study could not be conducted without the waiver of consent due to the need to administer the interventions as early as possible after the onset of cardiac arrest. Cardiac arrest patients are unable to provide consent.

(5) The proposed investigational plan defines the length of the potential therapeutic window based on scientific evidence, and the investigator has committed to attempting to contact a legally authorized representative for each subject within that window of time and, if feasible, to asking the legally authorized representative contacted for consent within that window rather than proceeding without consent. The investigator will summarize efforts made to contact legally authorized representatives and make this information available to the IRB at the time of continuing review.

Cardiac arrest is a time-critical condition. Victims of cardiac arrest collapse unpredictably in a range of public and private areas. Paramedics must mobilize to the patient side, bringing all portable monitoring equipment, medications, airway and ventilation equipment. In addition, paramedics must coordinate all resuscitation tasks, including delivery of chest compressions, insertion of intravenous line, and administration of medications. Documentation of clinical care and execution of trial procedures are difficult under these conditions.

Since this is an immediately life-threatening situation, it will not be possible to obtain consent prior to or at the actual time of treatment. A script describing the study will be provided to a recognized LAR on scene, when feasible, but it is acknowledged that the acute circumstances may only rarely if ever afford such an opportunity. We will make every effort to contact legal representatives after admission to the hospital to notify them that the patient was enrolled in a randomized trial and provide an opportunity to withdraw from further participation. Although OHRP regulations do not require that written informed consent be obtained once the patient is enrolled for continued

participation, the local IRB will determine whether a signed document is desirable for continued participation.

If legal representatives are not immediately available, research personnel will attempt to contact the subject's legal representative as soon as feasible and a summary of these efforts will be documented in the patient's chart. If the subject becomes competent during the study period then he/she will be approached by research personnel for notification of enrollment.

(6) The IRB has reviewed and approved informed consent procedures and an informed consent document consistent with Department of Health and Human Services (HHS)-Office for Human Research Protections (OHRP) Sec. 46.116 and 46.117 of 45 CFR Part 46.

All procedures and notification/consent forms will be approved by the Institutional Review Board (IRB) of each study site prior to the onset of the trial.

(7) Additional protections of the rights and welfare of the subjects will be provided, including, at least:

- **i. Consultation (including, where appropriate, consultation carried out by the IRB) with representatives of the communities in which the clinical investigation will be conducted and from which the subjects will be drawn;**
- **ii. Public disclosure to the communities in which the clinical investigation will be conducted and from which the subjects will be drawn, prior to initiation of the clinical investigation, of plans for the investigation and its risks and expected benefits;**
- **iii. Public disclosure of sufficient information following completion of the clinical investigation to apprise the community and researchers of the study, including the demographic characteristics of the research population, and its results;**
- **iv. Establishment of an independent data monitoring committee to exercise oversight of the clinical investigation; and**
- **v. If obtaining informed consent is not feasible and a legally authorized representative is not reasonably available, the investigator has committed, if feasible, to attempting to contact within the therapeutic window the subject's family member who is not a legally authorized representative, and asking whether he or she objects to the subject's participation in the clinical investigation. The investigator will summarize efforts made to contact family members and make this information available to the IRB at the time of continuing review.**

(i) In U.S, centers, community consultation as outlined by the local IRB will be undertaken prior to IRB approval. Since the population eligible for enrollment includes all citizens in the study regions it will not be possible to target any particular small group. The community consultation plan for each study site will have to be individualized to fit the IRB requirements. Appendix 5 contains an example of a community consultation plan that has been used in another study of this nature. Feedback from the community will be obtained by research personnel regarding any concerns they may have about potential enrollment. If requested, bracelets will be made available that could be worn by members of the community who do not want to participate.

(ii) & (iii) Public disclosures will be performed both prior to study enrollment and at the completion of the study in the form of multimedia press releases organized by the Resuscitation Outcomes Consortium. These will include plans for the study including potential risks and benefits and a summary of the results of the study upon completion. In the event that the press releases are not widely circulated, advertisements will also be placed in local papers describing the study.

(iv) An independent data monitoring committee will exercise oversight of the study as described below.

(v) We expect that all patients who meet the enrollment criteria will be unconscious. Any delay in medical care that would be required for the paramedic to attempt to obtain consent from the patient's legal guardian would be life threatening. Thus it will not be feasible to attempt to obtain informed consent during the initial therapeutic window. However, a brief written script describing the study (Appendix 5, Section 29.4) will be developed for presentation to a LAR on-scene by the prehospital provider, when feasible, giving an opportunity to object to study enrollment. Because the acute circumstances of cardiac arrest rarely if ever afford even such a limited opportunity without compromising patient care in process, determine if or when presenting this script is feasible, in light of these safety considerations, will be left to the clinical discretion of the provider..

The local ROC investigator will provide information about the emergency research study to the patient or their representative at the earliest feasible opportunity after administration of the intervention and given an opportunity to withdraw from further participation. Once the patient reaches the hospital no further research interventions will occur except for data collection. The local IRB will determine whether a signed document is desirable for continued participation. In the event that a patient or their family withdraws from ongoing participation survival from hospital discharge will be ascertained from publically available sources.

29. Appendix 5 – Example Patient Notification Forms for Use Under Exception from Informed Consent for Emergency Research

29.1 Notification Forms (for initially surviving Subjects)

Title of Research:
Pragmatic Trial of Airway Management in Out-of-Hospital Cardiac Arrest

IRB Protocol #: **[XXXXXXX]**

Investigator: **[XXXXXXX]**

Sponsor: The National Institutes of Health
The University of Alabama at Birmingham
University of Washington

Please note that the term "you" used in this document refers to either you or your family member

You had a cardiac arrest and survived. The Emergency Medical System (EMS) providers quickly started cardiopulmonary resuscitation (CPR). This includes breathing for you by inserting a tube (an airway tube) into your windpipe and pumping on your chest. They may have applied electricity to your heart, often referred to as defibrillation, to try and restore a normal heart beat. You were enrolled in a research study conducted by [institution] through the Resuscitation Outcomes Consortium (ROC). ROC is the largest prehospital clinical research consortium in the world, focusing on research in the areas of prehospital cardiopulmonary arrest and severe traumatic injury. The purpose of this notification form is to give you information about this study. You may ask questions about the purpose of the research, why you were enrolled, the possible risks and benefits, your rights as a research participant, and anything else about the research or this form that is not clear. We will give you a copy of this form for your records.

Cardiac arrest is an extreme emergency during which the patient will die within a few minutes if treatment is not begun immediately. Patients in cardiac arrest are unconscious and unable to discuss their treatment, and any time taken to discuss their treatment with family robs the patient of immediately starting life-saving measures. Because of this and because an airway tube must be inserted quickly after a cardiac arrest, you were entered into the study at the scene of the event. In this situation, oversight groups who are responsible for supervising and regulating such studies, including the United States Office for Human Research Protections (OHRP), have allowed us to enter people into the study without first obtaining written consent. This permission was granted only after informing and seeking input from the local community as required by an oversight group. These requirements may have included household surveys, press releases, and lectures to the medical and lay public.

There are also other safeguards in place for this research. First, the National Heart, Lung, and Blood Institute of the National Institutes of Health (NIH) which is supporting this research, had an independent Data and Safety Monitoring Board to review the research to make sure it was scientifically sound. The DSMB also monitors the results of the research during the course of the study to be sure of patient safety. Finally, a local group associated with the University of [ ] is also monitoring the research.

Purpose of the Research

The purpose of this study is to determine whether one of two airway tubes that are currently being used in the prehospital setting is better than the other. The two tubes being compared are 1) the “endotracheal tube,” which is a plastic breathing tube inserted into the airway (windpipe), and 2) a “supraglottic airway”, which is a tube inserted into the esophagus (the opening to the throat and stomach, where you swallow), to block off the esophagus so that air only goes into the lungs. Both types of tubes are used by EMS providers, but prior studies have not shown whether one works better than the other.

EMS providers treat cardiac arrest by performing cardiopulmonary resuscitation (CPR). This includes pushing on the chest with the heel of their hands to help the blood move, and if needed, helping a person to breathe by inserting a tube through the mouth into the throat so that air is able to reach the lungs. When a person cannot breathe on his or her own, an airway tube allows oxygen to reach the lungs and be circulated throughout the body during CPR.

The EMS providers who cared for you when you had your cardiac arrest are participating in a research study to see if we can determine whether one airway tube is better than another. Because of the seriousness of your illness and the immediate need for treatment, the providers were unable to ask for your permission to participate in the study. However, we told the public about this study before it started. Also, the [name of institutional] Institutional Review Board reviewed this study before it started and gave us permission to enroll subjects without their consent. The study is being supervised locally by Dr. [full name] of [institution]. This study is being conducted in six regional community centers within the US, and will enroll over 3,000 persons.

Explanation of Procedures

In this study, all participating EMS providers are using the same airway tube for a period of time, and then will switch to the other tube for the same amount of time. You had one of these two airway tubes inserted as part of this study. When EMS personnel cared for you, they performed all the procedures they would have normally performed. In the event that the EMS personnel were not able to use the airway tube being studied, they would have used an alternate method, such as a different type of airway tube.

After you were admitted to the hospital, all of the treatments for your cardiac condition were determined by your physicians, and this study did not interfere with such treatment in any way. Airway tubes inserted by EMS personnel may be removed or replaced at the doctor’s discretion.

As part of this study, we will record some information about you. We will collect information from the EMS agency about your medical condition when they arrived, and what care they administered to you. We will also collect information from your hospital stay about the care you received here, any reports from operations or other procedures you had (such as X-rays or other imaging), what medications you were given, and how long you stayed in the hospital.

Risks and Benefits of the Research

If there is any benefit to you from being in the study, it has already occurred. You will receive no further benefit from being in this study. However, your participation in this study will benefit society if we are able to show whether one of the two airway tubes being studied has better results.

Patient safety is carefully monitored and recorded for any complications from study treatments. As is possible with any medical intervention, there are risks involved with having an airway tube inserted; however, since this study uses two airway tubes that are currently being used by EMS providers, being enrolled in this study does not add additional medical risks.

The risks of insertion of an airway tube include unsuccessful airway insertion, multiple insertion attempts, airway tube misplacement (tube is inserted incorrectly), inadequate ventilation (tube does not allow enough air passing into the lungs), vomiting after airway insertion, cuts, scrapes, or perforations to the soft tissues inside the mouth of throat, injury to teeth, and a collapsed lung.

Alternatives

The best known emergency care for cardiac arrest was given to you by the EMS personnel. Cardiac arrest is normally treated, in part, with the use of airway tubes, including the types used in this study.

Confidentiality

Your confidentiality will be respected; information that discloses your identity will not be released without your consent unless required by law or regulation. Research records and medical records identifying you may be inspected in the presence of the investigator or his designate by representatives of the Research Outcomes Consortium (ROC), the National Institutes of Health (NIH), Office for Human Research Protections (OHRP), and the Institutional Review Boards (IRB – the committee that protects the rights of research subjects) of [institution]. Records that identify you by name or initials will not be allowed to leave the research offices of the investigators. Information about your condition will be sent to the ROC study Coordinating Center at the University of Washington in Seattle, WA. All information sent to the Coordinating Center is coded and no personal identifiers such as name, phone number, or address are included.

You have been assigned a specific code number. A key linking you to the code number is kept in a secure location and will be available only to the investigators and research staff at this institution. The study enrollment log is password protected and stored on the [explain site-specific data storage]. This study is expected to be completed by 2019. The data will be stored as per the [institution]'s IRB requirements for a period of [x] years and only then will the paper documents be shredded and data destroyed on-site.

Results from this study, without personal identity information, may be reported in scientific meetings, articles or other appropriate communication. If during the course of the study, new information becomes available, we will provide it to you. A description of this clinical trial will be available on http://www.ClinicalTrials.gov, as required by U.S. Law. This website will not include information that can identify you. At most, the website will include a summary of the results. You can search this website at any time.

[Include site-specific billing information]

Voluntary Participation and Withdrawal

You may withdraw from further participation in this study at any time after receiving this notification without penalty or loss of benefits to which you are otherwise entitled by telling us or by contacting the investigators at [phone number of local investigator]. We will, however, keep the

data collected up to the date that you withdraw yourself so that we are able to report on the safety of the study.

Cost of Participation

There will be no cost to you for participation in the research. The costs of your standard medical care will be billed to your insurance company in the usual manner.

Payment for Research-Related Injuries

[local institution], The University of Alabama at Birmingham, and the National Institutes of Health have not provided for any payment if you are harmed as a result of taking part in this study. If such harm occurs, treatment will be provided; however, this treatment will not be provided free of charge.

Significant New Findings

[Local PI] and [his/her] staff will provide any significant new findings that develop during the course of the study that may affect your willingness to continue in the research study.

Questions

[Include local institution's required language for contacting the local PI with questions about the study, and information to contact the local IRB about "rights as a research participant," or with concerns or complaints about the research]

Legal Rights [Need for this section will vary by institution.]

[You are not waiving any of your legal rights by signing this document.]

Signatures [Need for this section will vary by institution.]

[Your signature below indicates that you agree to continue to participate in this study. You will receive a copy of this signed consent form.]

29.2 Next of Kin Notification Letter (for family of subjects who did not survive)

Date
To the Family of (insert patient's name)
Address
Address
Dear Family Member:

We understand that this letter may come at a time that is difficult for your family, and we offer our condolences for your loss. We are aware a death is often an unexpected event and may have devastating personal consequences.

Your family member experienced a cardiac arrest and was transported to [institution]. We want to assure you that your family member received the best medical care currently practiced for treatment of cardiac arrest. In addition to the standard treatments for cardiac arrest, your family member was entered into a research study called "[Pragmatic Trial of Airway Management in Out-of-Hospital Cardiac Arrest]". We are writing you now to inform you that this occurred. This was done without their consent because they required emergency care that could not be delayed to allow adequate consideration of informed consent. No further action on your part is required. We are including with this letter, a description of the study. In addition to receiving the study intervention, research data and routine clinical information was collected as part of the study. Your family member's personal information will remain confidential. You may request that we not collect any further information on your family member by calling the number listed below; however, we cannot discard any information we collected prior to your request.

Your loved one's participation in this study will contribute a great deal to better understanding how we can improve the treatment of patients who have a cardiac arrest. Apart from our sharing this information with you, you will receive no further contact from study personnel.

This research is overseen by the Institutional Review Board (IRB) of [institution]. They oversee the safety of subjects in medical research. This study was described to the general population through the media in newspapers, radio and television. Community opinion was sought, and community leaders and citizens agreed that this study was needed. The ROC (Research Outcomes Consortium), located at [institution], is part of the largest Prehospital clinical research consortium in the world, focusing on research in the areas of Prehospital cardiopulmonary arrest and severe traumatic injury. If you have any questions about the research study, please feel free to contact me or my staff at the number listed below. If you have any questions about your family member's rights as a research participant, or concerns or complaints about the research, you may contact the [institution's name, name of IRB, phone number, and hours of operations]. You may also call this number in the event the research staff cannot be reached or you wish to talk to someone else.

We apologize for this intrusion. Please accept our sincere condolences to you and your family.

Kindest regards,

[PI]

29.3 Study Information Sheet (accompanies Next of Kin Notification Letter)

Title: Pragmatic Trial of Airway Management in Out-of-Hospital Cardiac Arrest

Your family member was treated for a cardiac arrest by Emergency Medical Services (EMS) personnel, and at [institution]. When he/she was being treated at the scene and in the emergency department, he/she was unconscious and/or unable to reliably talk with us about his/her wishes. During this time your family member was enrolled in a study called “Pragmatic Trial of Airway Management in Out-of-Hospital Cardiac Arrest”.

An Institutional Review Board (IRB) at [local site] has given us permission to do this study in which subjects are enrolled without their consent. This process is called exception from informed consent for emergency research.

During a cardiac arrest, cardiopulmonary resuscitation (CPR) is performed by EMS personnel. This includes breathing for the patient by inserting an airway tube into the windpipe, and pumping on the patient’s chest. The purpose of this study is to determine whether one of two airway tubes that are currently being used is better than the other. The two tubes being compared are 1) the “endotracheal tube,” which is a plastic breathing tube inserted into the airway (windpipe), and 2) a “supraglottic airway”, which is a tube inserted into the esophagus (the opening to the throat and stomach, where you swallow), to block off the esophagus so that air only goes into the lungs. Both types of tubes are used by EMS providers, but prior studies have not shown whether one works better than the other.

The study is being done throughout the United States and Canada by an emergency medicine network called the Resuscitation Outcomes Consortium (ROC), of which Dr. [local PI] is a member. The ROC is the largest prehospital clinical research consortium in the world, focusing on research in the areas of prehospital cardiopulmonary arrest and severe traumatic injury. This study is funded by a grant received from the U.S. National Institutes of Health.

Your family member’s participation in this study will help us to better understand treatment of cardiac arrest and how we can improve the care of future cardiac arrest patients. Our study evaluates the usual care and procedures routinely performed by Emergency Medical Service (EMS) Agencies throughout the United States and Canada. If you have any questions, concerns, or complaints about the research or a research related injury including available treatments, you may contact Dr. [PI] or [his/her] staff and they will be glad to answer any of your questions. Their number is [local site PI’s number] .

[Insert instructions for after-hours calls, as well as for questions about your rights as a research participant, and also for concerns or complaints about the research. Include office hours]

A description of this clinical trial will be available on http://www.ClinicalTrials.gov, as required by U.S. Law. This Web site will not include information that can identify you. At most, the Website will include a summary of the results. You can search this Website at any time.

29.4 Script for EMS Notification of Next of Kin

Emergency Provider Script for Verbal Notification of Exception from Informed Consent

**TITLE OF RESEARCH:**	Pragmatic Trial of Airway Management in Out-of-Hospital Cardiac Arrest
**IRB PROTOCOL:**	[Local information]
**INVESTIGATOR:**	[Local information]
**SPONSOR:**	National Institutes of Health (NIH)<br>University of Alabama at Birmingham<br>University of Washington

Instructions:

While at the scene, when the participating emergency medical system (EMS) provider determines the patient has met the study inclusion and exclusion criteria, the following information may be provided as feasible to the conscious and alert patient, legally authorized representative, and/or family member of the patient who has experienced a cardiac arrest.

The purpose of the study information script is to attempt to provide an opportunity for verbal objection to study enrollment or to offer the opportunity to decline further participation, if already enrolled, only if the situation is both safe and does not adversely affect patient care and/or transport. The acute circumstances should only rarely, if ever, afford such opportunities.

EMS providers will be trained to attempt notification only in situations when feasible to conscious or alert patient, legal representative or family member.

Script:

1. You (or your loved one) appear to have suffered a cardiac arrest and needs treatment to open the airway to introduce oxygen to the body to protect the brain and body from oxygen deprivation.

2. I want to let you know that we **(EMS Agency Name)** are conducting a research study that tests two different types of breathing tubes that are commonly used by EMS personnel for cardiac arrest patients.

3. Treatment needs to be done quickly, but we want to give you the option of not being enrolled in the study.

4. If you say no we will treat you (or your loved one) according to our standard protocols. Someone will give you more information when we reach the hospital.

29.5 Sample Community Consultation Plan

The following is an example community consultation plan from a prior interventional trauma study that we implemented with the Resuscitation Outcome Consortium IRB protocol, F120425003 – Pragmatic, Randomized Optimal Platelet and Plasma Ratios (PROPPR). Participating sites will develop individualized community consultation plans for the trial.

A. Goal

The community consultation process is designed to allow the public to provide input before the study begins. Community consultation also provides an opportunity to learn more about community members' understanding of the research and how they weigh the potential benefits and risks associated with the proposed study. This requires a clear presentation of the purpose of the study, the treatment, the potential risks and benefits of the study, along with the best available estimates of the results.

B. Objectives

- 1. Communicate with a variety of audiences and special interest groups with the target population being 15-45 years of age for traumatic injury.
- 2. Remind the community of the overall goal to improve emergency care (i.e., improve the public's health).
- 3. Educate the community that clinical research is vital to improving health care and saving lives and help them understand why emergency exception from informed consent is needed.
- 4. Describe the proposed study and its relationship to current gaps in our knowledge about treating critically injured patients.
- 5. Assess the community's understanding of the proposed research, associated risks and benefits, and emergency trauma care research.
- 6. Solicit input from community members regarding the acceptability of the study and their concerns about the study.

C. Message

We need the community's help to begin this important medical research. Members of our community need to provide their opinions on the study and the unique nature of the emergency exception from informed consent before the studies can begin. We encourage dissemination of information to the community and feedback from the community. A task force of ROC staff, IRB representatives and members of the public should initiate and encourage this important two-way communication through websites, newsletters and press releases.

This vital research depends on the combined efforts of the research staff and the trauma care providers at UAB. They have joined forces to improve emergency trauma care in our community.

By working with a consortium, we can obtain scientifically valid results that help identify more effective trauma treatments in less time.

Patient safety is our highest concern. Before implementation, the study must be approved by three different panels of scientists not directly affiliated with the study. The study will also have independent and ongoing safety monitoring.

Eligible patients either receive current standard of care treatments, many of which are unproven in terms of benefit, or alternatives that have shown potential promise. The assignment of treatment will occur by chance (i.e., assignment will be random; by chance). No eligible patient will receive preferential treatment.

Drawing on earlier studies, the researchers hope the therapy being studied will be better than current standard of care treatment. But, this promising treatment has ***not*** been proven to be better in a large clinical trial. This is the final step before a treatment becomes standard of care.

There is no additional charge to the eligible patient for participation in these studies.

The investigators recommend that people who don't want to participate in the study wear a clearly identifiable "Opt Out Of ROC Research" bracelet. It is not practical to pre-enroll study participants given the magnitude and duration of the study.

Our primary goal is community education and informing the public about exception from informed consent. The UAB trauma center is the only trauma center in Central Alabama. As such, patients from across central Alabama, who are transport by a participating EMS agency, will be eligible for enrollment if they meet the inclusion criteria and are transported to the UAB trauma center. Participating EMS agencies are: Air Methods (LifeSaver helicopters), Bessemer Fire Department, Birmingham Fire and Rescue, Center Point Fire District, Hoover Fire Department, NorthStar EMS, and Regional Paramedical Services in Walker and St. Clair county will be trained and participate in this study. Most eligible participants will comes from Jefferson, Shelby, Walker, St. Clair, Talladega, Chilton, Blount, Calhoun, Cullman, Etowah, Winston, Tuscaloosa, Greene, Pickens, Fayette, Bibb, Perry, Hale, Clay and Coosa and counties.

In conducting community consultation activities, the UAB ROC staff ensures that representatives from the communities involved in the research have an opportunity to participate in the consultation process. The UAB ROC staff proposes this multi-facetted approach for effective and broad-based community consultation and public disclosure activities so that members of as many different groups within the local ROC communities will have an opportunity for input.

1. Prior to start up: Mass Media

- Exposure in a large circulating newspaper.
- Exposure in networks affiliated with local television stations in conjunction with the University Media Relations department.
- Be available to answer questions raised by the public.

2. Prior to start up: Local infrastructure

- Provide designated information telephone for questions/comments.
- Provide an accessible website (www.uab.edu/arc) with additional information on the study (FAQ), opt-out methods, and contact information for the investigators and IRB.
- Keep a log of the questions and comments for subsequent IRB review.

3. Prior to start up: Local Media/Geographic Region/Site

- Announcement in the Birmingham News (at 1 month prior and start of data collection)
- A Random Digit Dialing survey of the eligible counties listed above will be solicited about this research study. The UAB Survey Research Unit will conduct the Random Digit Dialing survey and these results will be shared with the UAB IRB. Each county will be weighted based on the percent of population (see attached distribution).
- Attached is a manuscript described our previous experience using social media We propose utilize the same method and will develop a 90 character ad that will be an external link from Facebook, to the UAB ROC website (www.uab.edu/arc). This ad will be on the side bar and will appear only on Facebook users 15 years of age and older, and whom live within a 50 mile radius of Birmingham. According to Facebook, there are 592,240 Facebook users that meet that criteria. Our proposed ad (Listed below) would be displayed on the side-bar of this at-risk population of Facebook user accounts. If a user clicks on the ad, they are redirected to the UAB ROC website, where they can find information about this specific protocol (see FAQ, how to Opt-Out of the ROC studies, and contact information for the UAB ROC office). Using Google Analytics software, we can provide quantifiable information to the IRB about the number of visitors to each webpage, including the number of visitors to the study description page, and opt-out page.
- Be available to answer questions raised by the public.

4. During the study period: Mass and Local Media/Geographic Region

- Periodic (≈6 months) updates of study progression to media.
- Be available to answer questions raised by the public.

5. Completion of the study period: Mass and Local Media/Geographic Region

- Coordinated media press release with the NIH, and UAB media relations.
- Posting the study summary on the Alabama ROC website.
- Be available to answer questions raised by the public.

Opt-Out of the ROC studies

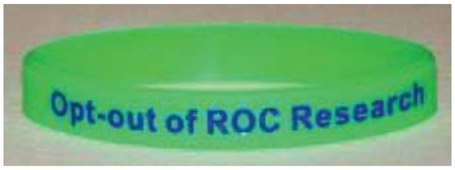

We respect the wishes of members of our community that do not wish to be enrolled in the ROC research clinical trials. If community members do not want to participate in these research trials, they can request to Opt-Out. We offer a silicone bracelet with the words "Opt-out of ROC Research" engraved on it. Community members will have to wear this bracelet during the time this study is being conducted beginning now – through the year 2015.

Even if someone opts out, there is no guarantee that under emergency circumstances the bracelet will be attached to the person. The individual might still be enrolled in the study. In the unlikely event they are enrolled after they have requested not to be enrolled; we will not use any of the information collected, and all information pertaining to the enrollment will be destroyed.

Two Ways to get your Opt-Out of the ROC Research material.

- Contact or visit the ARC office at (205) 934-9532 or via email: alabamaroc@uabmc.edu

- Stop by the ROC research office and pick up an Opt-Out wrist band. Our office is located in the Kracke Building on the Campus of UAB, room 531. The address is 1922 7th Avenue South Birmingham, Alabama.

Facebook Advertisement

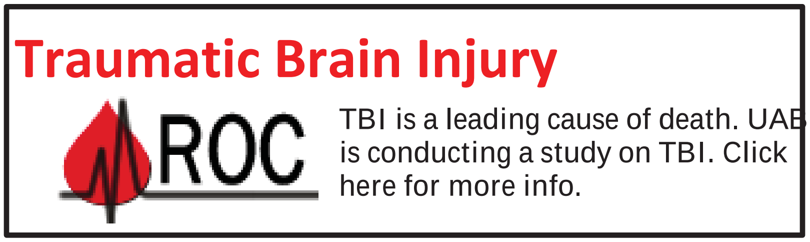

Supplement: Supplement 1. — Trial Protocol [file jamanetwopen-e2419274-s001.pdf]
