# Supplementary material for: Temporal Trends in End-Tidal Capnography and Outcomes in Out-of-Hospital Cardiac Arrest: A Secondary Analysis of a Randomized Clinical Trial
Source: JAMA Netw Open. 2024 Jul 5;7(7):e2419274. doi: 10.1001/jamanetworkopen.2024.19274 (PMC11227078; doi:10.1001/jamanetworkopen.2024.19274)
Supplement: Supplement 2. — eAppendix 1. Schematic of Time Points eAppendix 2. Variability in Capnography Change eAppendix 3. Stratified Analysis by Resuscitation Length of Time eAppendix 4. Generalized Estimated Equations to Account for Trial Design [file jamanetwopen-e2419274-s002.pdf]

Supplemental Online Content

Nassal MMJ, Elola A, Aramendi E, et al. Temporal trends in end-tidal capnography and outcomes in out-of-hospital cardiac arrest: a randomized clinical trial. *JAMA Netw Open.* 2024;7(7):e2419274. doi:10.1001/jamanetworkopen.2024.19274

eAppendix 1. Schematic of Time Points

eAppendix 2. Variability in Capnography Change.

eAppendix 3. Stratified Analysis by Resuscitation Length of Time

eAppendix 4. Generalized Estimated Equations to Account for Trial Design

This supplemental material has been provided by the authors to give readers additional information about their work.

© 2024 Nassal MMJ et al. *JAMA Network Open.*

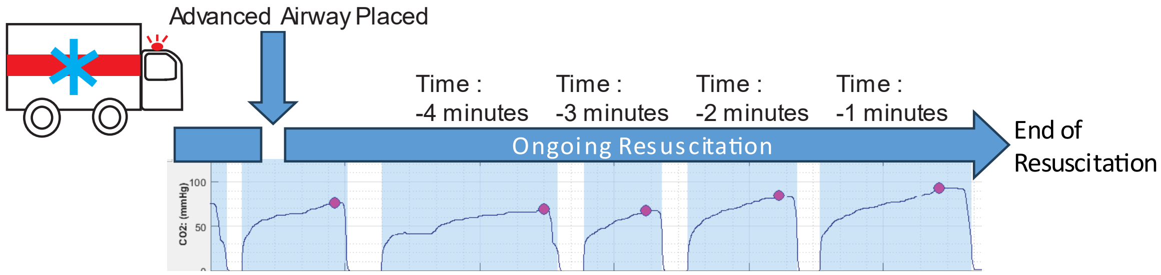

eAppendix 1: Schematic of Time Points

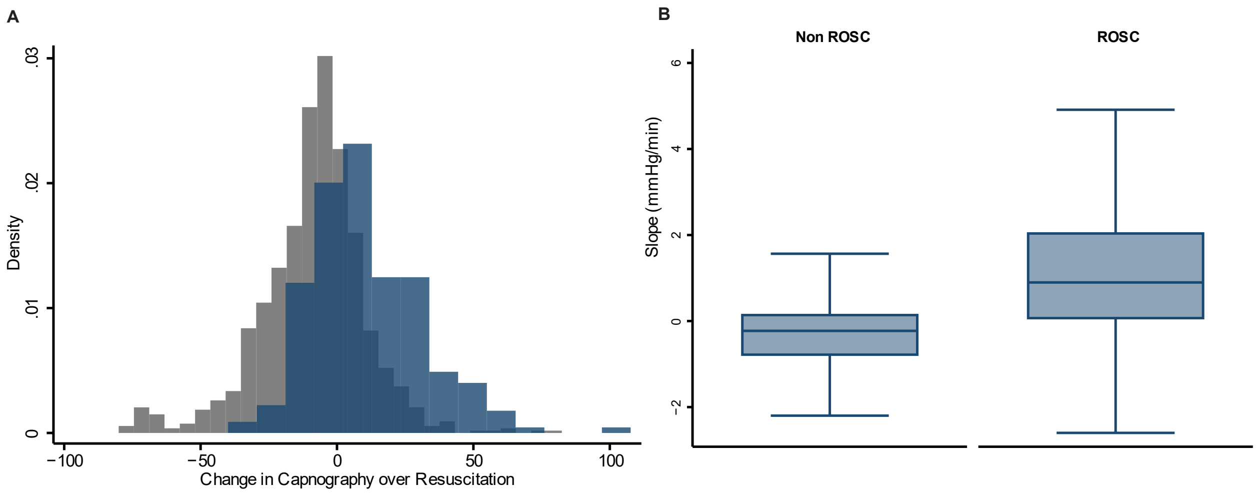

eAppendix 2: Variability in Capnography Change. Histogram plots of return of spontaneous circulation (ROSC, blue) change from initial to end of resuscitation event are shown. ROSC groups median change was 7.67mmHg (IQR:-2.74, 23.44 mmHg). NonROSC patients, depicted in grey, median change was -7.00mmHg (IQR:-18.61, 1.79 mmHg). Slope of capnography change is shown in B. ROSC slope median was 0.90mmHg/min (IQR: 0.05, 2.05 mmHg/min). NonROSC groups median slope was -0.23mmHg/min (IQR:-0.80, 0.15 mmHg/min).

© 2024 Nassal MMJ et al. *JAMA Network Open.*

Variable	ROSC (95% CI) < 10minutes Resuscitation N=229	ROSC (95% CI) > 10minutes Resuscitation N=762	Survival (95% CI) < 10minutes Resuscitation N=229	Survival (95% CI) > 10minutes Resuscitation N=762
Slope of $EtCO_2$	1.27 (1.12, 1.43)	1.53 (1.29, 1.83)	1.15 (1.03, 1.30)	1.42 (1.20, 1.67)
Shockable Rhythm	2.27 (0.88, 5.9)	1.71 (0.94, 3.11)	1.79 (0.72, 4.40)	4.38 (1.97, 9,72)
Age	0.98 (0.97, 1.00)	0.98 (0.97, 1.00)	0.98 (0.96, 1.00)	0.95 (0.52, 3.89)
Gender=male	0.99 (0.52, 1.89)	1.04 (0.60, 1.81)	1.20 (0.60, 2.43)	0.89 (0.39, 2.00)
Bystander Witnessed	7.29 (3.27, 16.28)	2.64 (1.47, 4.74)	7.06 (2.91, 17.13)	7.27 (2.43, 21.72)
EMS Witnessed	3.67 (1.39,9.64)	4.84 (2.22, 10.57)	4.86 (1.72, 13.75)	14.16 (3.84, 52.21)
Bystander CPR	1.06 (0.53, 2.16)	0.78 (0.44, 1.39)	0.98 (0.45, 2.14)	0.74 (0.30, 1.81)
Chest Compression Rate within 100-120	0.57 (0.27, 1.23)	2.27 (1.02, 5.04)	0.23 (0.10, 0.52)	2.07 (0.68, 6.28)
Chest Compression Depth within 5-6cm	0.83 (0.43, 1.58)	1.06 (0.49, 1.58)	0.75 (0.37, 1.52)	0.74 (0.34, 1.59)
Public Location	1.45 (0.41, 5.17)	2.46 (1.25, 4.82)	2.67 (0.80, 8.95)	2.42 (1.03, 5.72)

eAppendix 3: Stratified Analysis by Resuscitation Length of Time. EtCO2=End tidal capnography, CPR=Cardiopulmonary Resuscitation.

© 2024 Nassal MMJ et al. *JAMA Network Open.*

Variable	ROSC OR (95% CI)	Survival OR (95% CI)
Slope of $EtCO_2$	1.42 (1.3, 1.57) ***	1.26 (1.15, 1.39) ***
Age	0.99 (0.98, 1.0)*	0.97 (0.96, 0.99) ***
Gender=male	0.86 (0.59, 1.24)	0.81 (0.51, 1.29)
Shockable Rhythm	3.33 (2.21, 4.98) ***	5.4 (3.34, 8.75) ***
Bystander CPR	1.0 (0.72, 1.49)	1.07 (0.68, 1.69)
Bystander Witnessed	1.68 (1.29, 2.17) ***	1.91 (1.37, 2.63) ***

eAppendix 4: Generalized Estimated Equations to Account for Trial Design. *p<0.01, ***p<0.001

© 2024 Nassal MMJ et al. *JAMA Network Open.*
