# Supplementary material for: Temporal Trends in End-Tidal Capnography and Outcomes in Out-of-Hospital Cardiac Arrest: A Secondary Analysis of a Randomized Clinical Trial
Source: JAMA Netw Open. 2024 Jul 5;7(7):e2419274. doi: 10.1001/jamanetworkopen.2024.19274 (PMC11227078; doi:10.1001/jamanetworkopen.2024.19274)
Supplement: Supplement 3. — Data Sharing Statement [file jamanetwopen-e2419274-s003.pdf]

Data Sharing Statement

Nassal. Temporal Trends in End-Tidal Capnography and Outcomes in Out-of-Hospital Cardiac Arrest. *JAMA Netw Open*. Published July 05, 2024. doi:10.1001/jamanetworkopen.2024.19274

Data
Data available: Yes
Data types: Deidentified participant data
How to access data: NHLBI biolincc
When available: beginning date: 12-27-2019

Supporting Documents
Document types: None

Additional Information
Who can access the data: anyone requesting the data
Types of analyses: for any purpose
Mechanisms of data availability: Per NHLBI biolincc
